# Genetic Diversity and Population Structure of Bulgarian Autochthonous Sheep Breeds Revealed by Microsatellite Analysis

**DOI:** 10.3390/ani13111878

**Published:** 2023-06-05

**Authors:** Yanka Mihailova, Krasimir Rusanov, Mila Rusanova, Pavlina Vassileva, Ivan Atanassov, Vasil Nikolov, Elena G. Todorovska

**Affiliations:** 1 Agricultural Academy, 30 Suhodolska Str., 1373 Sofia, Bulgaria; jana661119@abv.bg; 2 Department of Agrobiotechnology, AgroBioInstitute, Agricultural Academy, 8 Dragan Tsankov Blvd., 1164 Sofia, Bulgaria; krusanov@abv.bg (K.R.); milagradeva@abv.bg (M.R.); pavlina_vassileva@abv.bg (P.V.); ivan_atanassov@abv.bg (I.A.); 3 Department of Livestock Sciences, Agricultural University (AU), 12 Mendeleev Blvd., 4000 Plovdiv, Bulgaria; v_nikolov@au-plovdiv.bg

**Keywords:** local sheep breeds, microsatellite markers, genetic diversity, population structure

## Abstract

**Simple Summary:**

Local, autochthonous sheep breeds are an important part of the socio-economic and production system in Bulgaria and Europe. Molecular characterization is a key factor for their preservation. In this study, we analyzed the genetic diversity and population structure of Bulgarian autochthonous sheep breeds using a set of 13 microsatellite markers. A total of 600 individuals from 50 flocks representing 12 breeds were included in the study. Our results showed that two of the breeds—Local Stara Zagora/SZ/ and Local Karnobat/MK/—were homogenous in terms of their genetic structure and could be easily differentiated from each other and from all other breeds. At the same time, all of the remaining breeds were an admixture with a heterogenous genetic structure, with some exceptions where occasional flocks were relatively homogenous within some of the breeds. Our study showed that it is necessary to implement proper management practices and effective sheep breeding strategies in Bulgaria to preserve the autochthonous breeds, maintain the genetic diversity, and prevent the erosion of the breed purity. The applied set of microsatellite markers could be further used as an effective molecular tool for the monitoring and development of such programs and strategies.

**Abstract:**

This study attempts to provide a deeper insight into the current genetic status of 12 Bulgarian autochthonous sheep breeds using microsatellite (SSR) markers. A total of 600 individuals from 50 flocks were analyzed using a panel of 13 SSR markers. In total, 228 alleles were found in the studied microsatellite loci. The mean number of alleles, the effective number of alleles, and the polymorphic information content (PIC) values per locus were 17.54, 5.250, and 0.799, respectively. The expected heterozygosity (He) for all breeds ranged from 0.70 to 0.82. The within-population heterozygote deficit (Fis) varied from −0.03 to 0.1, reflecting significant levels for 10 of the 12 breeds. The average genetic differentiation (Fst) was 0.046, revealing a low discrimination between the breeds. The genetic distance, principal coordinate analysis, and the structure analysis showed that two of the studied breeds—Local Stara Zagora/SZ/ and Local Karnobat/MK/—were the most distinct sheep populations. The Bayesian clustering approach suggested poor breed differentiation for the remaining 10 sheep breeds. The results suggest that proper management strategies and specific breeding policies need to be implemented in Bulgaria to avoid the intermixing of breeds and to reduce the erosion of breed purity observed in some breeds.

## 1. Introduction

The efficient management, conservation, and sustainable utilization of local sheep breeds is a key factor for successful and sustainable sheep breeding and farming, especially in countries with traditional mountain shepherding or sheep farming in areas with conditions that are less favorable for livestock. Bulgaria has a long and well proven tradition of sheep farming in areas with diverse climate and environmental conditions. Sheep breeding in Bulgaria involves mainly autochthonous breeds, which are an essential part of sheep farming in the country. The autochthonous sheep breeds are well adapted to the local conditions; possess high resilience to extreme climate conditions; resistance to diseases; and are of important historical, socioeconomic and cultural significance for the country. The loss of any local breed entails the loss of both genetic diversity and specific traits, which can hardly be restored again. The necessity to conserve genetic diversity in farm animals was recognized and ratified by the Convention on Biological Diversity (CBD) in 1993 [1]. Local breeds are a valuable genetic reservoir for the improvement of existing breeds and for the development of new breeds. Local breeds are also an important tool in organic animal husbandry. The topic is especially relevant in the context of the globally uniform selection criteria in breeding programs of commercial breeds and intensive selection, which leads to reduced genetic diversity in populations and increased levels of inbreeding and inbreeding depression.

The genetic diversity in farm animals is constantly decreasing, regardless of the measures taken on a European and global scale to preserve it. In Bulgaria, there has been a reverse trend owing to the good traditions in the protection of local genetic resources, which began in the 1970–1980s, and the strong support of the state in the last two decades. This led to a dramatic increase in the population size of some breeds over a short period of time. New purebred herds are being included under the control of breeding organizations. However, there are concerns that some non-typical representatives of the breeds, as well as crosses with introduced rams, may also be included. This is contrary to the aim of preserving given genetic resources by preserving and maintaining their unique qualities. Breed differentiation is still based solely on phenotype, which is often not effective because some breeds have similar exterior features. This calls for more precise methods for determining the affiliation and differentiation of local sheep breeds in Bulgaria.

There have not been systematic molecular studies covering a large number of sheep breeds in Bulgaria up until now, only small-scale molecular investigations including a few autochthonous sheep breeds and/or a few microsatellite markers [2,3,4,5,6]. Among the DNA marker systems used today, microsatellites (simple sequence repeats (SSR)) and SNP (single nucleotide polymorphism) have proven to be the most applicable markers for paternity testing; gene diversity assessment; analysis of the genetic structure and inbreeding; QTL mapping; and in population and evolutionary genetics in a range of animal species, including sheep [7,8,9].

At the end of the 1980s, the number of sheep in Bulgaria was nearly 10 million, belonging to more than 20 local sheep breeds, but now their number is less than 1 million [10]. This drastic reduction in the sheep population in Bulgaria has threatened the sustainable conservation of local sheep genetic resources and has led to the extinction of some sheep breeds such as Rilomonaster and Svishtov sheep. In 2021, 10 breeding organizations carried out breeding activities with 119,586 sheep of 19 autochthonous breeds approved by the Ministry of Agriculture (former Ministry of Agriculture, Food, and Forests (MAFF)) in Bulgaria. They all place emphasis on preserving the native sheep breeds as genetic resources and overcoming the risk of extinction by increasing their population size. This requires in-depth study of the local breeds at a genomic level, which is in line with the global Food and Agriculture Organization (FAO) directives for the conservation of the global genetic diversity to achieve sustainable agriculture and food security for the growing human population worldwide [11]. Such studies will give a better picture of the genetic diversity of local breeds, also helping to establish their genetic identity and outlining some conservation strategies.

The present study aimed to characterize the genetic diversity and population structure of Bulgarian autochthonous sheep breeds based on genotyping with microsatellite markers. The study included animals representing different flocks from 10 local sheep breeds farmed in different regions of the country. The microsatellite data for two additional local breeds, previously reported by Mihailova [3], were also included in the data pool and analyzed to gain an overall evaluation of the genetic diversity and structure of the Bulgarian autochthonous breeds. How the obtained data can be further utilized for the management and conservation of these sheep breeds in Bulgaria is discussed. 

## 2. Materials and Methods

### 2.1. Ethical Statement

All of the experimental procedures were reviewed and approved by the Animal Research Ethics Committee of the Bulgarian Food Safety Agency (BFSA) (Identification code 154 Art. 381 of the Law on Veterinary Activity) in accordance with European Union Directive 86/609.

### 2.2. Selection of Breeds for Genotyping

Ten Bulgarian local sheep breeds were selected for genotyping based on the analysis of the current state and trends of change, amount of research, geographical principle, and degree of threat: Local Stara Zagora/SZ/; Central Stara planina/SSP/; Duben/DAB/; Central Rhodope/SR/; Koprivshtitsa/KOPR/; Karakachan/KARA/; Local Karnobat/MK/; Replyan/REP/; Sakar/SAK/; and Breznik/BREZ/. Among them, SSP, DAB, KOPR, REP, and SAK were not included in previous molecular studies. 

The breeds were of a different origin: MK, KARA, REP, SSP, SAK, and SR sheep originate from Tsakel; DAB, KOPR, and BREZ are Tsigai type; while SZ is an intermediate form of the cross between the ancient Tsigai and Tsakel [12]. SR, KARA, MK, and SAK are short-tailed sheep, whereas SZ, SSP, DAB, KOPR, REP, and BREZ are long-tailed sheep. The fleece color is pigmented (SSP, DAB, KOPR, and MK), white (SZ, REP, SAK, and BREZ), or of mixed type (SR and KARA) [13].

Among the Tsakel-type sheep in Bulgaria, KARA is the most typical and the most primitive representative of the coarse-wool breeds. It is highly adapted to harsh natural conditions and a nomadic lifestyle. REP and MK are selected mainly for the production of wool and meat. REP is used for the production of the famous Bulgarian Chiprov carpets, while MK lambs were highly valued and sought after in the market in Tsarigrad, the Ottoman Empire (present-day Istanbul, Turkey), in the 19th century and earlier [14]. SZ is selected mostly for dairy production. The remaining local breeds are used for the production of wool, meat, and milk. 

### 2.3. Sampling and DNA Extraction

**Figure 1:**
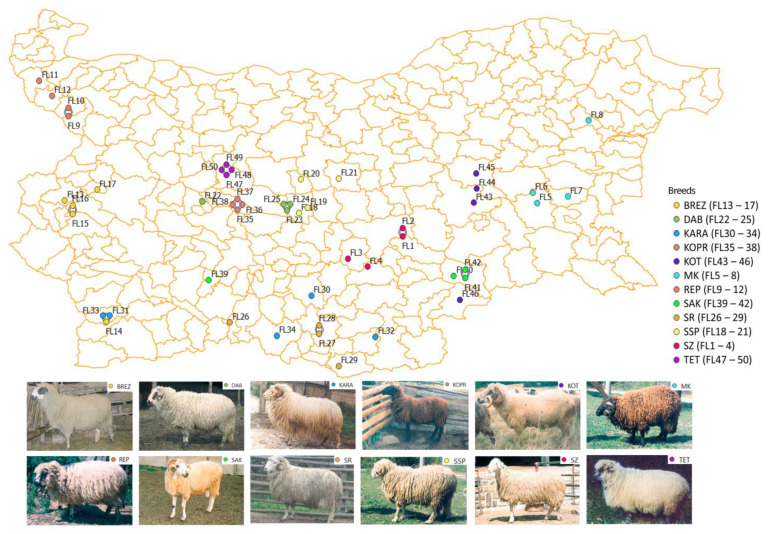
Geographical location of the 50 flocks from 12 Bulgarian sheep breeds included in the study: Breznik (BREZ), Duben (DAB), Karakachan (KARA), Koprivshtitsa (KOPR), Kotel (KOT), Local Karnobat (MK), Replyan (REP), Sakar (SAK), Central Rhodope (SR), Central Stara planina (SSP), Local Stara Zagora (SZ), and Teteven (TET). Flocks shown as connected are located in one and the same settlement.

A total of 504 animals from 42 flocks were used in the study. Twelve animals were sampled from each flock in accordance with the Food and Agriculture Organization (FAO) instructions given in Section C of Part 4 of The State of the World’s Animal Genetic Resources for Food and Agriculture [15]. The animals were selected by experts from the breeding organizations working with the respective breed together, with the experts of the control body “Animal Breeding and Breeding Executive Agency” (IASRG) of the Ministry of Agriculture, Bulgaria. The animals were unrelated and were phenotypically representative of each breed. Ten of the studied breeds were presented by 4 flocks (48 animals), while 2 of them—BREZ and KARA—were presented by 5 flocks (60 animals). The microsatellite data for two local breeds of Tsakel type —Teteven/TET/ and Kotel/KOT/—represented by 8 flocks and 96 animals were reported previously by Mihailova [3], and were also included in this study to obtain more comprehensive information about the overall genetic diversity and structure of the sheep breeds in Bulgaria. The geographical distribution and location of the sampled flocks are shown in Figure 1 and Supplementary Table S1. For some of the breeds, samples were also taken from flocks outside of their main area of distribution; for example, FL 14 of BREZ and FL 46 of KOT. The habitats of SSP, DAB, KOPR, TET, and KOT are located in close proximity to the Balkan Mountains (Stara Planina mountain), which splits Bulgaria into two zones (northern and southern) with different climatic conditions.

Blood samples were collected from the vena jugularis into vacutainer tubes (Venoject^®^, Terumo, Lakewood, CA, USA) with an anticoagulant (K_2_EDTA) and were immediately put into a cooler bag and transported to the laboratory, where they were stored at −20 °C prior to DNA extraction.

The DNeasy Blood and Tissue Kit (QIAGEN, Hilden, Germany) was used to isolate genomic DNA with the help of a QIAcube system for automated DNA isolation (QIAGEN, Hilden, Germany). The concentration of the isolated DNA was measured spectrophotometrically with a Qubit 3.0 Fluorometer (Thermo Fisher Scientific Inc., Waltham, MA, USA) using a Qubit assay probe kit (Thermo Fisher Scientific Inc., Waltham, MA, USA). The quality and integrity of the DNA samples was determined using a 1% agarose gel electrophoresis. Visualization of the nucleic acids was done by staining with GelRed^®^ (Biotium, Fremont, CA, USA) on a Gel Documentation System WGD 30S (Witeg Labortechnik GmbH, Wertheim, Germany). DNA was stored at −20 °C until PCR amplifications.

### 2.4. Microsatellite Amplification

Genotyping was performed with 13 microsatellite markers (D5S2, INRA5, MAF65, OarAE129, OarFCB11, INRA23, OarFCB20, McM527, CSRD247, HSC, MAF214, OarCP49, and INRA63) recommended by FAO, https://www.fao.org/dad-is (accessed on 4 June 2023) and the International Society for Animal Genetics (ISAG), https://www.isag.us/Docs/AppGenSheepGoat2017.pdf (accessed on 4 June 2023). The microsatellite markers were selected based on their level of allelic diversity, according to the recommendations for use in genotyping and paternity tests. In order to provide the widest possible range, markers were chosen to cover 18 of the 54 chromosomes (*Ovis aries*, 2n = 54), resulting in genomic coverage of about 33% of the total chromosome number. The markers included in the study are shown in Supplementary Table S2.

All PCR reactions were carried out in a volume of 20 μL containing 20 ng gDNA, 10 μL 2× MyTaq™ HS Mix (Meridian Bioscience, Newtown, OH, USA), 10 pmol of each primer (Forward and Reverse), and ultrapure water up to the final volume of the reaction mixture. The markers included in the study and multiplex information are shown in Supplementary Table S2.

All PCR reactions were performed on an Axygen™, MaxyGene II Thermal Cycler (Corning Inc., New York, NY, USA) using the following PCR conditions: For multiplexes B and D: 1 cycle of denaturation at 95 °C for 12 min, followed by 31 cycles each consisting of 20 s at 95 °C, 1 min at 63 °C, 1 min at 72 °C, and 5 min elongation at 72 °C. For multiplexes A and C: 1 cycle of denaturation at 95 °C for 10 min, followed by 31 cycles each consisting of 30 s at 95 °C, 30 s at 55 °C, 1 min at 72 °C, and 5 min elongation at 72 °C. For amplification of INRA63, the PCR program included 1 cycle of denaturation at 95 °C for 12 min, followed by 31 cycles each consisting of 20 s at 95 °C, 1 min at 58 °C, 1 min at 72 °C, and a final elongation step at 72 °C for 5 min.

### 2.5. Fragment Analysis

Fragment analysis was performed on a 3130 Genetic Analyzer (Thermo Fisher Scientific Inc., Waltham, MA, USA) equipped with 36 cm long capillaries. LIZ 500 (Thermo Fisher Scientific Inc., Waltham, MA, USA) was used as an internal standard to determine the length of the amplified microsatellite alleles. Electropherograms were analyzed using a GeneMapper ™ v4.0 (Thermo Fisher Scientific Inc., Waltham, MA, USA).

### 2.6. Statistical Analysis

The calculation of the number of alleles per locus (Na), the effective number of alleles (Ne), the mean number of alleles (Mn), the expected (He) and the observed (Ho) heterozygosity, Nei’s genetic distance between breeds, the number of rare alleles, F-statistics (Fis, Fit, and Fst), genetic flux (Nm), the Hardy−Weinberg equilibrium (HWE), analysis of molecular variance (AMOVA), and principle coordinate analysis (PCoA) were performed using GenAlEx v 6.50 [16]. A Neighbor-Joining dendrogram based on Nei’s genetic distances was built using FAMD 1.31 [17]. The polymorphic information content (PIC) for each SSR marker was determined using PowerMarker v 3.25 software [18]. Structure v 2.3.4 [19] was used to analyze the genetic structure of the populations, where an admixture was used as an ancestry model, Length of Burnin Period was set to 100,000, and the Number of MCMC Reps after Burnin was set to 200,000. The number of presumed clusters (K) was set from 1 to 16. Ten simulations were run for each value of K. Parallelization of Structure 2.3.4 calculations was achieved using EasyParallel [20]. The most probable K was determined using the Delta K method by Evanno et al. [21] with the help of a Structure Harvester [22]. The different iterations at a single K value were combined using Clumpak [23]. 

## 3. Results

### 3.1. Population Genetic Diversity Based on Microsatellite Markers

The fragment analysis of the amplified PCR products in the whole set of 600 animals of 12 local Bulgarian sheep breeds found polymorphism in all 13 autosomal microsatellite loci (Table 1). A total of 228 alleles were identified, ranging in number (Na) from 8 at locus D5S2 to 32 at locus OarCP49, with a mean of 17.54.

The average number of alleles per locus (Mn) varied from 5 in locus OarAE129 to 15.333 in locus OarCP49, with an average number of alleles in the studied 13 SSR loci of 9.994.

The average effective number of alleles (Ne), which is an important indicator of the intrabreed genetic diversity, was 5.25. The expected heterozygosity (He) ranged from 0.636 at locus OarAE129 to 0.842 at locus HSC, with an average of 0.784 across the populations for the 13 analyzed microsatellite loci. The observed heterozygosity (Ho) fluctuated between 0.516 at locus OarAE129 to 0.837 at locus OarCP49 with a mean of 0.759 for the whole population consisting of 12 sheep breeds. The He value exceeded that of Ho, which is an indication of heterozygous deficiency. The absence of a heterozygous deficiency was observed in 5 loci, where the values of He and Ho were the same or very close to each other (MAF65 and INRA63) or He was lower than Ho (McM527, MAF214, and OarCP49).

The polymorphic information content (PIC) was higher than 0.5 for all of the analyzed markers, ranging from 0.618 for marker OarAE129 to 0.87 for marker HSC, which is an indication that all loci were highly polymorphic. PIC was also high for OarCP49, INRA23, and OarFCB20 with values of 0.864, 0.858, and 0.858, respectively. The average PIC for the 13 microsatellite markers was 0.799.

The mean values of the fixation indices Fis, Fit, and Fst in the studied loci were 0.034, 0.078, and 0.046, respectively. The inbreeding coefficient (Fis) varied from −1 to +1 [24]. It is an indicator of a tendency to kinship between individuals in a population and is considered to be the main reason for deviation from the Hardy−Weinberg equilibrium (HWE). The average value of Fis in the 13 analyzed loci was 0.034, indicating a low level of inbreeding in the studied population of 12 sheep breeds. The highest level of heterozygous deficiency was observed in locus OarAE129 (Fis = 0.189), and the lowest in locus McM527, where a negative value was found (Fis = −0.005).

The value of Fit, which is used for measuring the heterozygosity loss of the individuals relative to the overall population, was 0.078, indicating about 8% overall deficit of heterozygous individuals in the sheep population. The mean value of Fis was 0.034, indicating an absence of heterozygous deficit in the studied loci.

The global breed differentiation evaluated by Fst ranged from 0.034 (MAF65) to 0.058 (INRA5 and OarAE129). Fst characterizes the level of genetic differentiation between populations based on the frequency of alleles in the respective microsatellite loci and varies from 0 to 1. In the present study, the mean Fst index was 0.046, which is evidence that the genetic differentiation between the studied breeds is low. This value shows that the general genetic variation is mainly due to differences between individuals (95.4%) and only 4.6% was as a result of differences between breeds. 

### 3.2. Genetic Diversity between Bulgarian Autochthonous Sheep Breeds

The results of the genetic diversity analysis among the studied breeds (Table 2) show that the lowest total number of alleles and number of alleles per locus in the breeds were scored for MK (82 and 6.31, respectively) and the highest ones were scored for KOT (149 and 11.46, respectively). The effective number of alleles ranged from 3.80 (MK) to 5.97 (DAB), with a mean of 5.25. 

The observed heterozygosity (Ho) varied from 0.66 (SZ) to 0.81 (DAB and SR), while the expected heterozygosity (He) varied from 0.70 (SZ) to 0.82 (DAB). In all breeds, with the exception of MK and SR, the average values of He did not exceed Ho. The coefficient of inbreeding (Fis) varied from −0.03 (MK) to 0.1 (KARA). Fis was higher than 0.05 only in two breeds, KARA and TET, while in the others, except for SSP, it was lower than 0.05. These values indicate that there is a risk of increased inbreeding only in KARA and TET. FAO categorizes a breed as vulnerable when ΔF is between 0.5 and 1% [25]. The present study is a good starting point for the evaluation of the breeds’ vulnerability by studying the change in the inbreeding coefficient over several generations. 

Twelve out of the thirteen microsatellites showed significant departures from the Hardy−Weinberg equilibrium in the whole population (Supplementary Table S3). Deviations from HWE were observed in all of the breeds. In SZ, REP, KARA, KOPR, and TET, there was deviation from HWE in four loci. BREZ deviated for three markers (OarFCB11, *p* < 0.05; McM527, *p* < 0.01; and OarCP49, *p* < 0.001). SSP, DAB, SR, and SAK deviated for two markers, while MK and KOT deviated for one marker (OarFCB20, *p* < 0.01 and CSRD247, *p* < 0.001, respectively). The most pronounced departure (*p* < 0.001) from HWE was observed in four loci, namely D5S2 (REP and DAB), INRA5 (KOPR), INRA23 (SZ), and CSRD247 (TET), which might be explained by the uncontrolled mating in the history of the breeds rather than the presence of null alleles. However, there was no available pedigree to use in the estimation of null alleles or for an unbiased estimation of inbreeding (identity by descent rather than identity by state). 

### 3.3. Frequency of the Alleles in the Studied Loci

Establishing the frequency of alleles at each locus is the basis for the assessment of genetic diversity (He), as well as the level of informativeness of the microsatellite markers used. 

The frequencies of the alleles in the studied loci by breeds are shown in Supplementary Figure S1. A breed-specific allele with a frequency of >5% was found only in REP (8.3%) in locus OarFCB1. Although this allele (146 bp) was found in only three of the four studied flocks, it was not identified in the remaining 11 breeds. Therefore, it could contribute to the differentiation of these flocks from the fourth one. 

Alleles with a frequency between 1% and 5%—referred to as rare alleles—were also found in 10 of the studied breeds (Table 3). These alleles contributed additionally to the differentiation of breeds, albeit to a very small extent, and are the basis for the high allelic diversity observed. They could be used successfully in paternity testing. Such alleles were found in four loci of SR (INRA5, OarFCB20, CSRD247, and INRA63) and KOT (INRA5, McM527, CSRD247, and INRA63); in three loci of BREZ, SZ, and SAK; in two loci in REP, SSP, KOPR, and TET; and in one locus in DAB. The highest number of rare alleles was observed in locus INRA63 (REP, BREZ, SR, SAK, KOT, and DAB) and MAF214 (BREZ, SSP, SAK, and TET). Very rare or unique alleles with a frequency of <0.01 were found only in BREZ and KOPR breeds. 

It is noteworthy that most of the rare alleles were found in two or three flocks of a breed and, although they cannot be used to identify the breed, they are a main reservoir for increasing the allelic (genetic) diversity in the sheep population.

In addition, as seen from Supplementary Figure S1, the most common alleles (i.e., evolutionarily the oldest ones) were represented with different frequencies in the different breeds. Alleles with a frequency of >0.5 were found in several loci in six of the studied breeds. In SZ, such alleles were found in five loci: MAF214 (allele 187 bp, 0.677), OarAE129 (allele 139 bp, 0.615), INRA5 (allele 220 bp, 0.594), D5S2 (allele 188 bp, 0.542), and OarFCB11 (allele 136 bp, 0.531). However, the frequency of the above-mentioned alleles in the other breeds was up to 14 times lower than that in SZ.

Alleles with a frequency above 0.5 were identified in two loci in MK, including OarAE129 (allele 151 bp, 0.585) and MAF214 (allele 187 bp, 0.583). In the rest of the breeds, such alleles were found only in one locus, including MAF214 (allele 187 bp, 0.635) in TET, OarAE129 (allele 151 bp, 0.508) in BREZ, OarAE129 (allele 151 bp, 0.578) in KARA, and MAF214 (allele 187 bp, 0.510) in SSP.

There were also other major alleles with frequencies lower than 0.5, but their values also varied widely in the breeds. Although they do not belong to population-specific alleles, differences in their frequency among the breeds contributed to their differentiation.

### 3.4. Genetic Differences between the Local Sheep Breeds

#### 3.4.1. Pairwise Comparison of Fst between Breeds

Fst between each pair of breeds was calculated in order to analyze their degree of differentiation (Table 4). The highest Fst was found between SZ and KARA (0.065), SZ and MK (0.063), SZ and REP (0.059), as well as between SZ and TET (0.056). Values of Fst > 0.05 were also observed between SZ and KOT (0.053), SZ and SSP (0.052), and SZ and KOPR (0.051). Similar values were found between SZ and the remaining four studied breeds. Relatively high Fst (>0.04) was also observed when comparing MK with BREZ, KARA, KOPR, and TET, but slightly lower between MK and the remaining breeds. 

Fst among the other breeds was <0.02, except between TET and BREZ (0.021), TET and DAB (0.02), TET and KARA (0.027), and TET and SAK (0.02). The lowest Fst was observed between KOT and KARA (0.010), as well as between KOT and SSP (0.011) and KOT and SR (0.011). 

#### 3.4.2. Genetic Distance between Breeds

The minimum Nei genetic distances were calculated based on the results obtained for the allele frequencies in the 13 microsatellite loci. The obtained values of genetic distances are presented in Supplementary Table S4.

The observed high values of genetic distance between SZ and KARA (0.437), SZ and REP (0.412), SZ and MK (0.362), and SZ and TET (0.362) correspond to the differences in the studied microsatellite loci at the genome level expressed as differences in the allele lengths, and, respectively, their frequencies. The genetic distances between SZ and the other breeds such as SSP, KOPR, DAB, SR, SAK, and BREZ were also significant (between 0.325 and 0.294). 

High values of genetic distances were also observed between MK and KARA (0.312), KARA and TET (0.282), and KARA and KOPR (0.278), while the distance between MK and the other breeds varied between 0.257 and 0.202.

The genetic distances between the remaining 10 breeds were significantly lower (<0.200). The lowest ones were between KOT and SSP (0.057), KOT and SR (0.052), and KOT and KARA (0.044).

#### 3.4.3. Genetic Structure and Genetic Relationships between Breeds 

**Figure 2:**
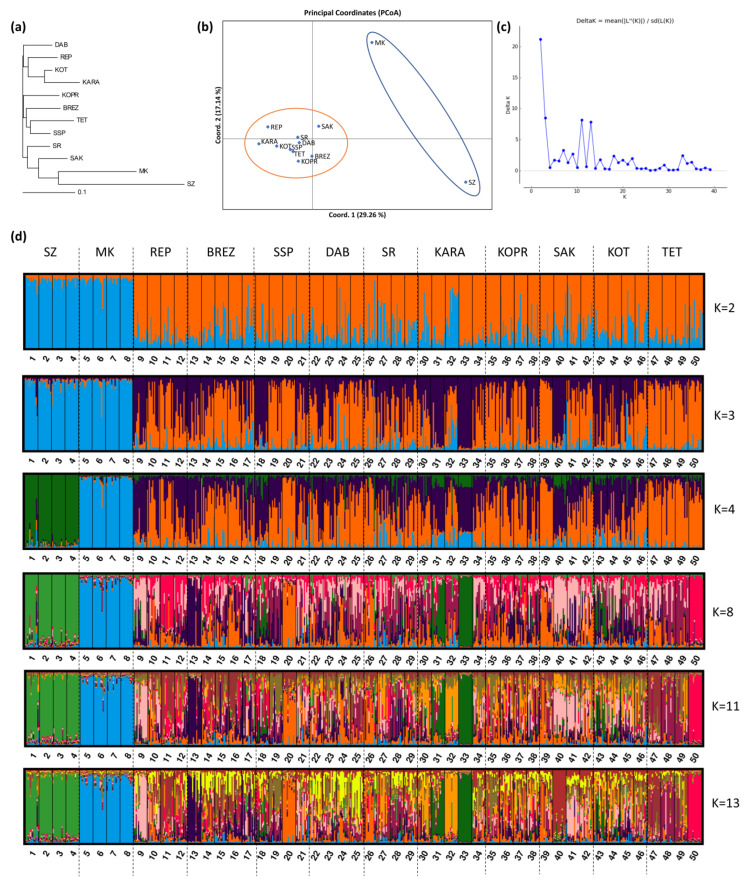
Analysis of the genetic structure and relationships between 600 individual animals from 50 flocks of 12 autochthonous Bulgarian sheep breeds: (**a**) Neighbor-joining dendrogram based on Nei’s genetic distances; (**b**) principal coordinate analysis; (**c**) estimation of the most probable number of genetic clusters using the delta K method by Evanno et al. [21]; (**d**) bar plots representing the genetic structure of the studied breeds at several K values, where black lines separate the flocks, while dotted vertical lines separate the breeds.

The Neighbor-Joining dendrogram (Figure 2a) constructed on the basis of Nei’s standard genetic distances showed three main clusters. Each of the clusters consisted of four sheep breeds. Cluster 1 included DAB, REP, KOT, and KARA; Cluster 2, KOPR, BREZ, TET, and SSP; and Cluster 3, SR, SAK, MK, and SZ. Genetically, SZ and MK were the most distant, while KOT and KARA were the most similar, as well as TET and SSP. 

The principal coordinate analysis (PCoA) identified two major groups of samples. They included MK and SZ on the one hand, and all other samples falling in the second group (Figure 2b). 

The genetic structure of the population represented by 50 flocks of the 12 studied autochthonous sheep breeds was determined using Structure v 2.3.4. The most probable number of genetic clusters, as determined by the delta K method of Evanno et al. [21], was two, three, eleven, and thirteen (K = 2, K = 3, K = 11, and K = 13) (Figure 2c). Figure 2d illustrates the genetic structure at K = 2, K = 3, K = 4, K = 8, K = 11, and K = 13 of the population of 600 sheep animals from the analyzed 50 flocks of the whole set of 12 breeds. The genetic structure at K=2 correlated well with the PCoA analysis. At K = 2, the studied population of 12 sheep breeds showed two main clusters. Cluster 1 included two breeds (SZ and MK), while the remaining 10 breeds fell into Cluster 2. The percentage of affiliation of individuals to the second cluster was high, which is evidence that these breeds have a common ancestor and are not clearly differentiated.

Evanno’s delta K method showed a few additional lower peaks, the highest of which was at K = 3 and K = 11, followed by K = 13, which indicated sub-structuring within the two main genetic clusters established at K = 2.

Figure 2d shows a process of separation of the two breeds SZ and MK at K = 4. In addition, the flocks of both breeds FL1-FL4 and FL5-FL8, respectively, showed a more pronounced homogeneous intra-breed structure than the other 10 breeds from the second genetic cluster at K = 2, whose structure is an admixture of the two clusters.

The low level of genetic differentiation of the breeds, with the exception of SZ and MK, showed that the phenotypic differences between the studied breeds were not accompanied by drastic changes at the genetic level, at least with respect to the studied loci. The breeds are characterized by high heterogeneity due to past and, in part, current gene flow as a result of animal exchange between breeds, which is evident at K = 4, K = 8, K = 11, and K = 13, or insufficient divergence of subpopulations from the original source.

Some fragmentation was observed within several of the breeds from the second genetic cluster at K = 2, as a result of geographical isolation and/or the use of heterozygous breeding rams. Such is the case with KARA, which formed two subpopulations within the breed. Two of the flocks of KARA (FL31 and FL33) with an area of distribution in South-West Bulgaria (villages Vlahi and Kresna, in the Blagoevgrad region) (Figure 1) and two flocks (FL30 and FL32) from the region of Asenovgrad and Momchilgrad (South Bulgaria), were differentiated from each other at K = 8. The animals from both pairs of flocks fell into different sub clusters within the breed, which was clearly expressed at K = 11 and K = 13. The flock from the region of Smolyan (FL34) with an area of distribution in the Rhodope Mountain, near Greece, showed minimum affiliation to the subclusters of the other two pairs of flocks (FL31 and FL33, and FL30 and FL32, respectively) of KARA. It seems that geographic isolation in combination with targeted selection led to a reduction in some alleles typical of KARA in flocks FL31 and FL33 located in South-West Bulgaria and to their pronounced genetic differentiation.

The individuals from flock FL20 of SSP were also differentiated from the other flocks of the breed at higher K values (K = 8, K = 11, and K = 13). The results were similar with flock FL9 of REP and flock FL13 of BREZ at K = 13 from the village of Nepraznentsi, Breznik, which were characterized by a more pronounced homogeneous structure in comparison with the rest of the flocks of both breeds. Similarly, flock FL50 of TET also showed differentiation from the other three flocks of this breed at K = 8.

Interestingly, the animals from some flocks of different breeds showed a similar percentage of affiliation to the respective sub clusters within the breed. The observed similarity can be attributed to the percentage of some shared evolutionarily old alleles from a common ancestor(s), their frequency among the breeds, genetic exchange (gene flow), and the current breeding strategies.

The differentiation of some flocks from the remaining flocks of a breed—as is the case with flocks FL31 and FL32 and flocks FL30 and FL33 of KARA, as well as flock FL50 of TET—could be explained by “the founder effect”.

## 4. Discussion

In the present study, 13 microsatellite markers were used to assess within and between breed genetic diversity and the population structure of 12 autochthonous sheep breeds in Bulgaria. The studied sheep population consisting of 600 individuals from 50 flocks showed a high level of genetic diversity, as indicated by the number of alleles, the level of heterozigosity, polymorphic information content (PIC), and other parameters related to genetic diversity (Table 1). The determined mean number of alleles (17.54 per locus) indicated high allele diversity in the entire sheep population. The set of microsatellite markers used in this study was very informative, as 11 out of 13 markers showed a high number of alleles (>12) and four markers (CSRD247, MAF214, OarCP49, and INRA63) identified more than 20 alleles. The mean number of alleles (Na) and the genetic variability of the Bulgarian local sheep population were similar to those reported for 11 Austrian sheep breeds (15.08 alleles/locus) [26], 10 indigenous Greek sheep breeds (14.5 alleles/locus) [27], Montenegrin sheep populations (13.5) [28], and for a breed grown in three areas in Western Anatolia (14.5 alleles/locus) [29]. A higher value for the mean number of alleles per locus (24.67) and the corresponding total number of alleles was obtained for 12 Algerian sheep breeds analyzed via 15 microsatellite markers [30], while lower values of the number of alleles per locus were reported in four indigenous Romanian sheep breeds (13.22 alleles/locus) [31], a sheep breed with an area of distribution in Albania and Kosovo (11.66 alleles/locus) [32], and three Albanian sheep breeds (11.23 alleles/locus) [33].

The effective number of alleles (Ne) is another important indicator of intra-breed genetic diversity. The average effective number of alleles (5.250) in our study suggested that all the sampled breeds had a high level of genetic variability. Similar results for Ne were reported for three Albanian sheep breeds [33]. Significantly higher values for this indicator (11.05, 10.57, and 7.3) were identified for Algerian sheep breeds [30], Turkish and Algerian indigenous breeds [34], and a native sheep breed raised in Western Anatolia [29]. 

Seven out of the 13 markers used in our study showed Ho > 0.8 ranging from 0.516 at locus OarAE129 to 0.837 at locus OarCP49, with a mean of 0.759 for the entire sheep population consisting of 12 sheep breeds. Similar mean Ho values (0.76) were reported for Turkish and Algerian sheep breeds [34], and considerably lower values (0.698) [32], (0.696) [27], (0.691) [31], (0.62) [35], (0.698) [26], and (0.523) [6] in sheep breeds from different parts of the Balkan Peninsula (Albania and Kosovo, Greece, Romania, and Bulgaria) and Europe (Austria and Hungary). Higher values of Ho were obtained in a Turkish breed of sheep (0.81) [29] and native Greek breeds bred on the island of Lesvos (0.837) [36]. There was heterozygous deficiency in all 25 microsatellite loci in a study of Austrian sheep breeds [26], similar to that found in previous studies of seven Bulgarian sheep breeds (Local Stara Zagora, Local Karnobat, Breznik, Elinpelin, Copper-red Shumen, Pleven black-headed, and Karakachan) [35] and another five Bulgarian sheep breeds (White Marishka, Patch-faced Maritza, Pleven black-headed, Stara planina, and Rhodope tsigai) [5]. 

The expected heterozygosity (He) was high in our study, varying from 0.636 (OarAE129 locus) to 0.842 (HSC locus), with an average of 0.784 across the entire population. Values of He close to those obtained in the present study were reported by Baumung et al. [26] (0.795). A higher Hе was obtained by Abdelkader et al. [30] (0.90), and lower He by Ligda et al. [27], Mastranestasis et al. [36], Hoda et al. [32], and Hoda et al. [33] (0.741, 0.733, 0.773, and 0.749, respectively).

There were nearly equal values of Ho and He (MAF65, INRA23, OarFCB11, McM527, and INRA63) in the present study, and for some loci, even higher values of Ho compared to He (MAF214 and OarCP49). This indicated that the genetic diversity of the studied population of sheep is high, but also that interbreeding occurs in the population. He exceeded Ho in several loci (D5S2, INRA5, OarAE129, OarFCB20, CSRD247, and HSC), indicating heterozygous deficiency.

The results (Table 1) showed that all of the studied loci are highly polymorphic and confirm the effectiveness of the selected set of SSR markers. The polymorphic information content (PIC) was higher than 0.5 for all of the analyzed markers with an average of 0.799, a value similar to that reported by Markovic et al. [28], but lower than the reported by Abdelkader et al. [30].

The within population inbreeding estimates or heterozygous deficiency within the whole population (Fis) was positive for most of the loci, with the exception of four loci (McM527, MAF214, OarCP49, and INRA63) with a mean value of 0.034. The negative value of Fis observed in some loci indicates a higher proportion of heterozygous individuals that could be explained by high gene flow between flocks of the breeds and the avoidance of mating related animals. The observed Fis value was similar to that found by Hoda et al. [33] (0.041) and Hoda et al. [32] (0.048) for sheep breeds from Albania and Kosovo, but lower than those previously reported for Bulgarian sheep breeds (0.22 [35] and 0.288 [5]), as well as for Austrian (0.054), Greek (0.60), Romanian, and Turkish sheep breeds (0.161) [26,27,31,34]. Low Fis values were reported by Yilmaz et al. [29] (0.03) and Abdelkader et al. [30] (0.032), and a negative one (−0.143) by Mastranestasis et al. [36].

The genetic differentiation over the loci was low (Fst = 0.046), indicating a high gene flow (Nm) between breeds, which ranged from 4.076 (INRA5) to 6.931 (OarFCB20) with a mean of 5.334. Gene flow (Nm) is an important parameter because it can substantially affect the level of genetic differentiation between breeds, especially for those that inhabit nearby areas with similar ecological conditions, regardless of their phenotypic differences as a result of selective pressure. A very low value of Nm (0.8) has been reported [32] as a result of the insignificant exchange of genes between the sheep populations distributed in Albania and Kosovo, explained by their long isolation since the state borders established after 1913 and the different breeding programs implemented. The observed low pairwise Fst values in the Bulgarian sheep population were likely as a result of genetic exchange between the local breeds due to insufficient selection control and incorrect exchange of animals, as discussed by Abdelkader et al. [30], Kdidi et al. [37], and Sassi-Zaidy et al. [38] for Algerian and Tunisian sheep breeds. Similar results for Fst have been reported for other sheep breeds from the Balkan Peninsula [4,35]. Low values of genetic differentiation have also been reported by Ligda et al. [27] (3.1%), Dudu et al. [31] (3.4%), Mastranestasis et al. [36] (2.1%), and Hoda et al. [33] (1.1%) for Greek, Romanian, and Albanian sheep breeds, as well as by Gaouar et al. [39] and Kandoussi et al. [40], for Moroccan (1.33 and 3.64%), Algerian (1.9%), and Tunisian (1.7% and 3%) sheep, but higher ones were reported by Baumung et al. [26] (8%) and Hoda et al. [32] (23.8%).

The genetic diversity between the studied 12 Bulgarian sheep breeds regarding the mean Na, Ho, and He showed high genetic diversity in the studied breeds, except for SZ and MK, which had, on average, only 8.15 and 6.31 alleles and Ho and He lower than 0.75. KOT, DAB, SAK, SR, and REP had the highest number of alleles per population (Na) and mean expected heterozygosity (He), indicating that they are the most genetically diverse breeds. The studies conducted by Hristova et al. [4,35] of the genetic diversity among seven local sheep breeds, including LZ, MK, BREZ, and KARA, showed a higher value of Na and He compared with our results for SZ and MK. This indicates a decreasing trend in the diversity in these two breeds during the last 10 years. Fis was positive in all breeds with the exception of MK, which indicates an excess of homozygotes and may also be a reason for the observed deviations from HWE in these breeds. However, the comparative analysis with previous studies [2,4,35] showed that the level of inbreeding has not increased in the autochthonous Bulgarian sheep breeds during the last 10 years. This is probably due to the increased size of the populations and the inclusion of new flocks under selection control, which allows for the more effective implementation of inbreeding avoidance schemes. Higher Fis values than those reported here were obtained for 10 Greek sheep breeds analyzed via 31 microsatellite markers [27], for five Bulgarian sheep breeds via 16 SSRs [5], but negative Fis values were reported in the studied flocks of the Greek Lesvos sheep analyzed via 11 microsatellite markers [36].

The present study demonstrates that only 4.6% of the total genetic variation in the Bulgarian sheep breeds is due to population differences. The results are similar to those obtained by Hristova et al. [4,35] and Chinkulov et al. [41], but higher than those reported for Greek (3.1% and 2.1%), Romanian (3.4%), and Albanian (1.1%) sheep breeds [27,31,33,36]. The low value of mean Fst observed in this study is an indication that the studied breeds are not differentiated enough. The lack of clear differentiation between the Bulgarian sheep breeds could be due to the geographic proximity and similarity in environment, but most likely the breeding practices based only on few phenotype characteristics typical for the breed [4,35]. Undoubtedly, with so many breeds located in a small area with overlapping regions of distribution that are not isolated and are geographically poorly differentiated—mostly on the Balkan Mountains—over the centuries there has been and still is an ongoing process of gene exchange. Moreover, all Bulgarian breeds belong to two types—Tsakel, Tsigay, or their crosses. Our study shows that only one allele could be referred as population-specific in REP, although with a low frequency (8.3%). The large number of low-frequency alleles (<5%) found in 10 of the breeds studied here is extremely suitable for tracking the dynamics of the genetic structure of the population and the direction of the genetic control as a result of factors of elimination or fixing of the unique alleles, increase or decrease in genetic diversity due to adaptation to new specific ecological and geographical conditions, the national selection guidelines, specific approaches of the farmers, the exchange and use of rams, etc.

The genetic structure analysis provides information needed to distinguish the breeds or populations by estimating the proportion that each individual or population carries from the genome of its parents or ancestors in order to assign individuals to any of the breeds or to define the level of homogeneity of populations [42].

The results of the genetic structure analysis, similar to those of the PCoA, show that the 12 Bulgarian autochthonous sheep breeds are clustered into two gene pools. SZ and MK are assigned to a separate gene pool. The most plausible explanation is that these breeds represent small and isolated populations of different exterior types [4,35]. Furthermore, this clear separation can help to promote their conservation and to implement breeding programs in accordance with the production, socio-economic, and cultural systems in Bulgaria. The second pool consists of the other 10 breeds, with a high level of overlapping probably due to their common ancestry, rearing in nearby geographic areas, and continuous gene flow between the populations, which is also visible at increasing K values. 

The study clearly shows that the genetic structure of autochthonous sheep breeds in Bulgaria has been significantly influenced by various factors including a large decrease (1990–2010) and subsequent increase (since 2010) in the population size. This is often accompanied by uncontrolled crossing among animals from different autochthonous breeds or with recently introduced highly productive foreign breeds, as well as carrying out breed selection without an appropriate breeding plan and methods for control. All this had led to a reduction in the genetic uniformity of the local sheep breeds and calls for a long-term policy and actions to preserve their genetic uniqueness in order to achieve sustainable agriculture and food security under ever-changing climatic conditions. Currently, the animal selection and reproduction management of the breeds in Bulgaria are based on the animal phenotype. This approach, however, is not sufficiently precise to preserve the unique genotype of the breed. In addition, the genetic processes that take place in the population, such as an increase or decrease in the level of inbreeding and the loss of genetic diversity cannot be evaluated on the basis of phenotype. Therefore, the effective management of sheep breeds needs an overall molecular-genetics characterization of the sheep populations and further monitoring of the changes in the genetic diversity and structure in order to develop and implement effective programs for “in situ” conservation of genetic resources, including the preservation of semen and embryos from selected animals. The present microsatellite characterization of a large part of the autochthonous breeds in the country provides a solid basis and essential monitoring to implement conservation programs and strategies for the preservation of Bulgarian local breeds and their further use for sustainable sheep farming.

## 5. Conclusions

This study is the first attempt to analyze the genetic diversity, population structure, and relationship of a large number of Bulgarian autochthonous sheep breeds among which five new ones, not investigated previously, i.e., Central Stara planina/SSP/, Duben/DAB/, Koprivshtitsa/KOPR/, Replyan/REP/, and Sakar/SAK/, using 13 microsatellite markers. The results revealed a high overall genetic diversity, but low genetic differentiation (4.6%) between the studied breeds, as well as a low level of inbreeding. The PCoA and Bayesian approach were effective at detecting the close genetic relationship among the studied breeds and their high level of admixture, except for the Local Stara Zagora/SZ/ and Local Karnobat/MK/ breeds. The low genetic differentiation between the breeds is the result of divergent management strategies, intermixing of breeds, and a lack of specific selection policies. The present study is a cornerstone for implementing proper management practices and designing effective breeding strategies to reduce the intermixing and erosion of the breed purity and develop effective “in situ” conservation programs in Bulgaria that require the introduction of measures, such as the use of proven rams and ensuring their frequent exchange between flocks. The results also showed that microsatellite markers are an appropriate tool for assigning animals/flocks to specific breeds and monitoring of the admixture processes where there is no strong control on the proper management of autochthonous sheep breeds.

However, further analysis based on medium or high-density SNP markers (50K or 600K SNP BeadChips) and the inclusion of some neighboring and other foreign sheep breeds are needed to obtain more comprehensive information about the genetic diversity and the place of Bulgarian autochthonous sheep breeds on a global scale.

## Figures and Tables

**Table 1:** Allele range, number of identified alleles per locus (Na), number of effective alleles (Ne), average number of alleles (Mn), observed (Ho) and expected (He) heterozygosity, polymorphic information content (PIC), F statistics in the studied simple sequence repeat (SSR) loci—intrapopulation coefficient of inbreeding (Fis), interpopulation coefficient of inbreeding (Fit), and coefficient of genetic differentiation (Fst)—and gene flow (Nm) in the studied loci.

Locus	Allele Range	Na	Mn	Ne	Ho	He	PIC	Fis	Fit	Fst	Nm
D5S2	188–202	8	6.083	3.410	0.664	0.700	0.689	0.052	0.097	0.047	5.064
INRA5	116–152	15	10.333	6.148	0.745	0.818	0.855	0.089	0.142	0.058	4.076
MAF65	121–147	13	7.750	3.980	0.744	0.744	0.737	0.000	0.034	0.034	7.047
OarAE129	139–319	9	5.000	2.819	0.516	0.636	0.618	0.189	0.237	0.058	4.042
OarFCB11	118–146	15	8.917	5.518	0.805	0.811	0.827	0.008	0.050	0.042	5.747
INRA23	196–224	15	10.583	6.429	0.815	0.822	0.858	0.008	0.064	0.056	4.205
OarFCB20	88–118	16	11.000	6.611	0.824	0.841	0.858	0.021	0.055	0.035	6.931
McM527	161–248	12	8.167	5.170	0.802	0.798	0.808	−0.005	0.034	0.038	6.245
CSRD247	209–265	24	11.500	5.586	0.807	0.819	0.837	0.014	0.053	0.040	6.058
HSC	181–303	20	11.667	6.531	0.771	0.842	0.870	0.083	0.125	0.045	5.274
MAF214	165–275	24	11.167	3.979	0.722	0.714	0.732	−0.011	0.043	0.054	4.406
OarCP49	72–140	32	15.333	6.438	0.837	0.833	0.864	−0.004	0.044	0.048	4.951
INRA63	157–213	25	12.417	5.632	0.810	0.809	0.832	−0.001	0.044	0.045	5.292
Mean		17.54	9.994	5.250	0.759	0.784	0.799	0.034	0.078	0.046	5.334
Total		228									

**Table 2:** Genetic diversity estimates among the studied 12 autochthonous sheep breeds. Total and mean number of alleles per population (Na), effective number of alleles (Ne), observed and expected heterozygosity (Ho and He), and inbreeding coefficient (Fis).

Breed	Number of Alleles	Na	Ne	Ho	He	Fis
SZ	106	8.15	3.85	0.66	0.70	0.04
MK	82	6.31	3.80	0.74	0.72	−0.03
REP	140	10.77	5.65	0.78	0.81	0.04
BREZ	139	10.69	5.01	0.76	0.78	0.04
SSP	140	10.77	5.68	0.76	0.80	0.05
DAB	136	10.46	5.97	0.81	0.82	0.01
SR	135	10.38	5.76	0.81	0.81	0.00
KARA	125	9.62	5.00	0.71	0.78	0.10
KOPR	138	10.62	5.20	0.77	0.79	0.03
SAK	137	10.54	5.88	0.79	0.81	0.02
KOT	149	11.46	5.86	0.79	0.81	0.04
TET	132	10.15	5.34	0.73	0.78	0.07
Mean	129.92	9.99	5.25	0.76	0.78	0.03

**Table 3:** Rare and unique alleles with a frequency of <5% and <1%, respectively, identified in the microsatellite loci in the analyzed breeds.

Breed	Locus	Allele		Breed	Locus	Allele	
		bp	Frequency			bp	Frequency
SZ	OarAE129	174	0.010	SR	INRA5	122	0.010
	MAF214	165	0.010		OarFCB20	88	0.021
		251	0.010		CSRD247	251	0.021
	OarCP49	105	0.010		INRA63	199	0.011
		138	0.010	SAK	INRA23	196	0.011
REP	INRA63	181	0.010		INRA63	175	0.010
	MAF65	147	0.031		MAF214	275	0.031
BREZ	MAF65	121	0.025	KOT	INRA5	116	0.010
	MAF214	243	0.033		McM527	248	0.010
	OarFCB11	118	0.008		CSRD247	265	0.010
	HSC	181	0.008		INRA63	209	0.010
	INRA63	197	0.025				
SSP	MAF214	241	0.010	TET	CSRD247	223	0.010
	OarSR49	140	0.031		MAF214	205	0.010
KOPR	OarAE129	319	0.009				
	OarCP49	72	0.008	DAB	INRA63	213	0.010
		114	0.010				

**Table 4:** Comparison of Fst between breeds.

Breed	SZ	MK	REP	BREZ	SSP	DAB	SR	KARA	KOPR	SAK	KOT
MK	0.063										
REP	0.059	0.037									
BREZ	0.049	0.040	0.017								
SSP	0.052	0.039	0.013	0.013							
DAB	0.049	0.036	0.012	0.014	0.013						
SR	0.049	0.034	0.012	0.015	0.012	0.012					
KARA	0.065	0.046	0.018	0.024	0.018	0.020	0.016				
KOPR	0.051	0.042	0.016	0.018	0.015	0.014	0.014	0.021			
SAK	0.048	0.034	0.016	0.020	0.016	0.015	0.013	0.023	0.018		
KOT	0.053	0.039	0.012	0.015	0.011	0.012	0.011	0.010	0.015	0.016	
TET	0.056	0.043	0.018	0.021	0.015	0.020	0.018	0.027	0.018	0.020	0.018

## Data Availability

The data presented in this study are available in the article and Supplementary Materials.

## Supplementary Materials

The following supporting information can be downloaded at: https://www.mdpi.com/article/10.3390/ani13111878/s1. Figure S1: Allele frequencies in 13 microsatellite loci based on analysis of 600 sheep from 12 Bulgarian local (autochthonous) breeds. Breed abbreviations: Local Stara Zagora/SZ/; Central Stara planina/SSP/; Duben/DAB/; Central Rhodope/SR/; Koprivshtitsa/KOPR/; Karakachan/KARA/; Local Karnobat/MK/; Replyan/REP/; Sakar/SAK/; Breznik/BREZ/. Table S1: Distribution of flocks of breeds across the country. Some of the flocks are distributed outside of their natural habitats. Table S2: Information for the used microsatellite markers and their inclusion in multiplex PCR reactions. na = data not available. Table S3: Hardy−Weinberg (HW) equilibrium test in the 13 analyzed microsatellite loci by breed. Table S4: Genetic distances between breeds according to Nei.
