# Supplementary material for: Genetic Diversity and Population Structure of Bulgarian Autochthonous Sheep Breeds Revealed by Microsatellite Analysis
Source: Animals (Basel). 2023 Jun 5;13(11):1878. doi: 10.3390/ani13111878 (PMC10252131; doi:10.3390/ani13111878)

**Supplementary Figure S1.** Allele frequencies in 13 microsatellite loci based on analysis of 600 sheep from 12 Bulgarian local (autochthonous) breeds. Breed abbreviations: Local Stara Zagora /SZ/; Central Stara planina /SSP/; Duben /DAB/; Central Rhodope /SR/; Koprivshtitsa /KOPR/; Karakachan /KARA/; Local Karnobat /MK/; Replyan /REP/; Sakar /SAK/; Breznik /BREZ/.

**Locus D5S2**


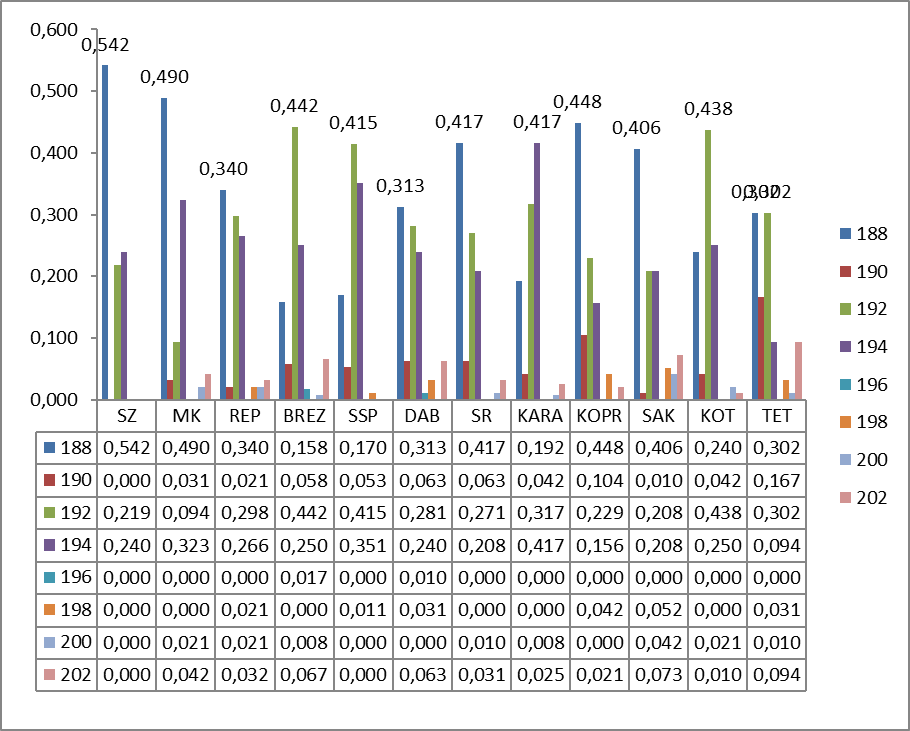


**Locus INRA5**


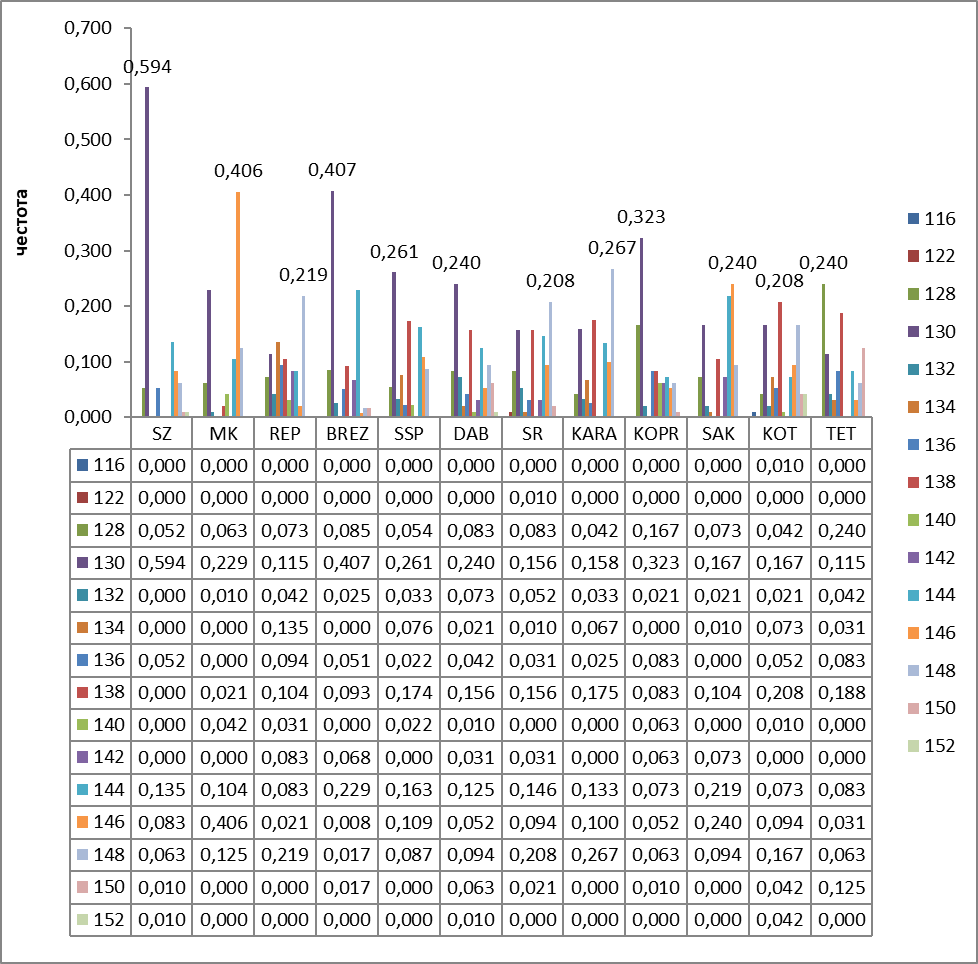


**Locus MAF65**


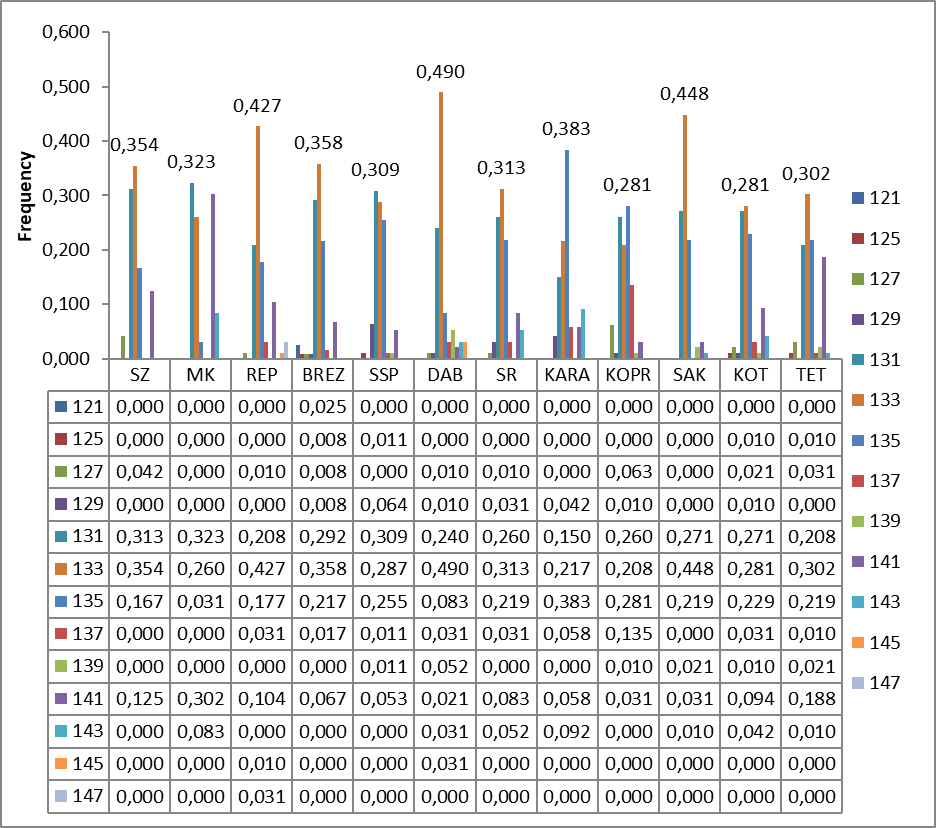


**Locus OarAE129**


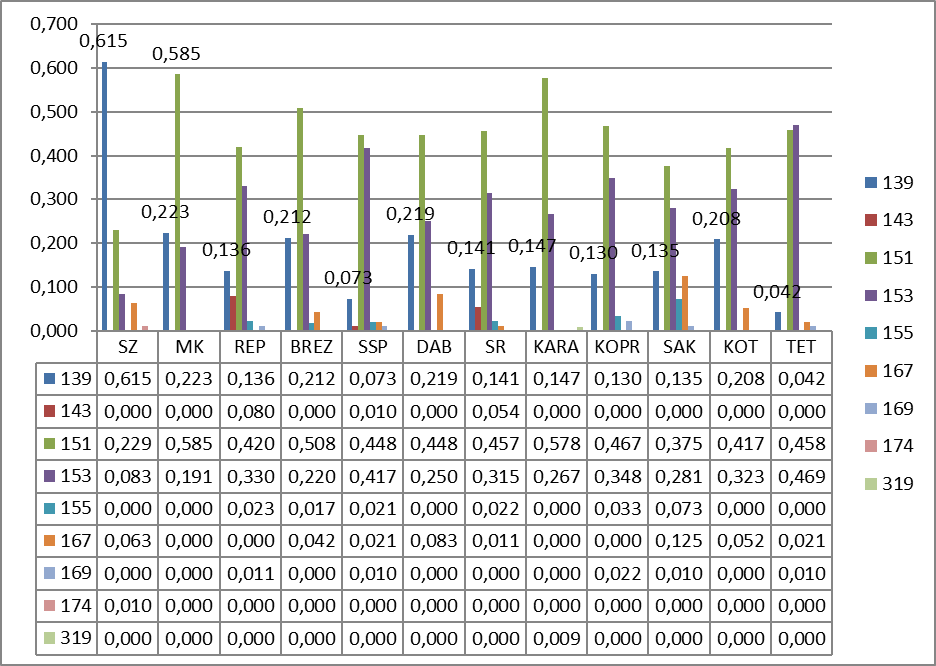


**Locus OarFCB11**


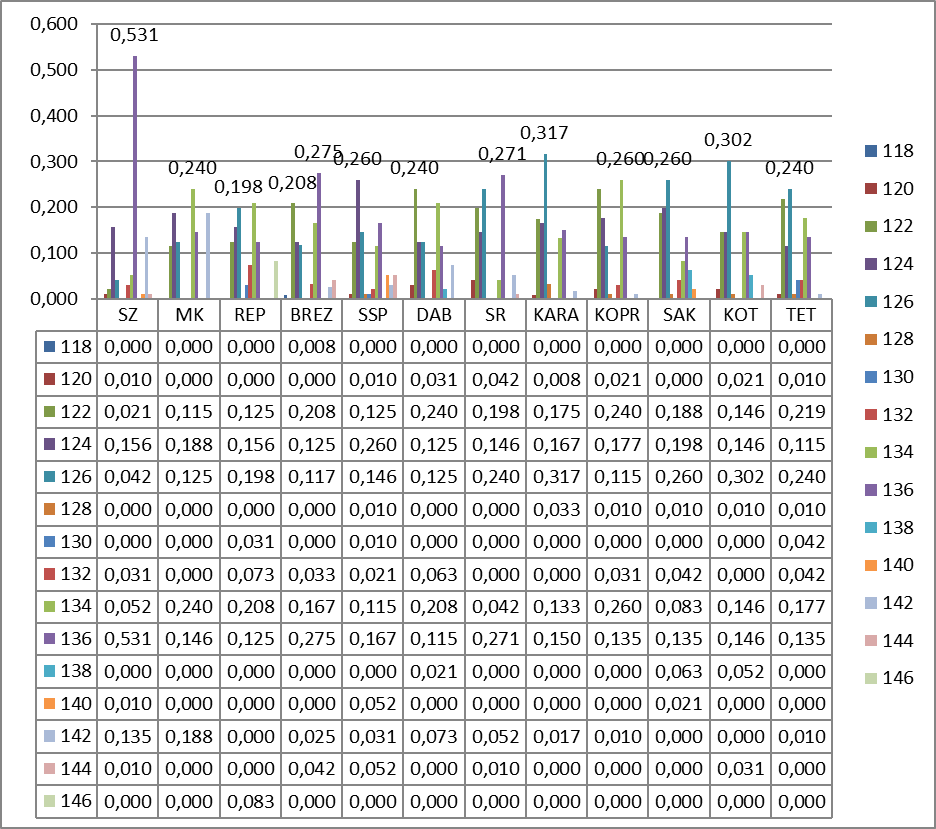


**Locus INRA23**


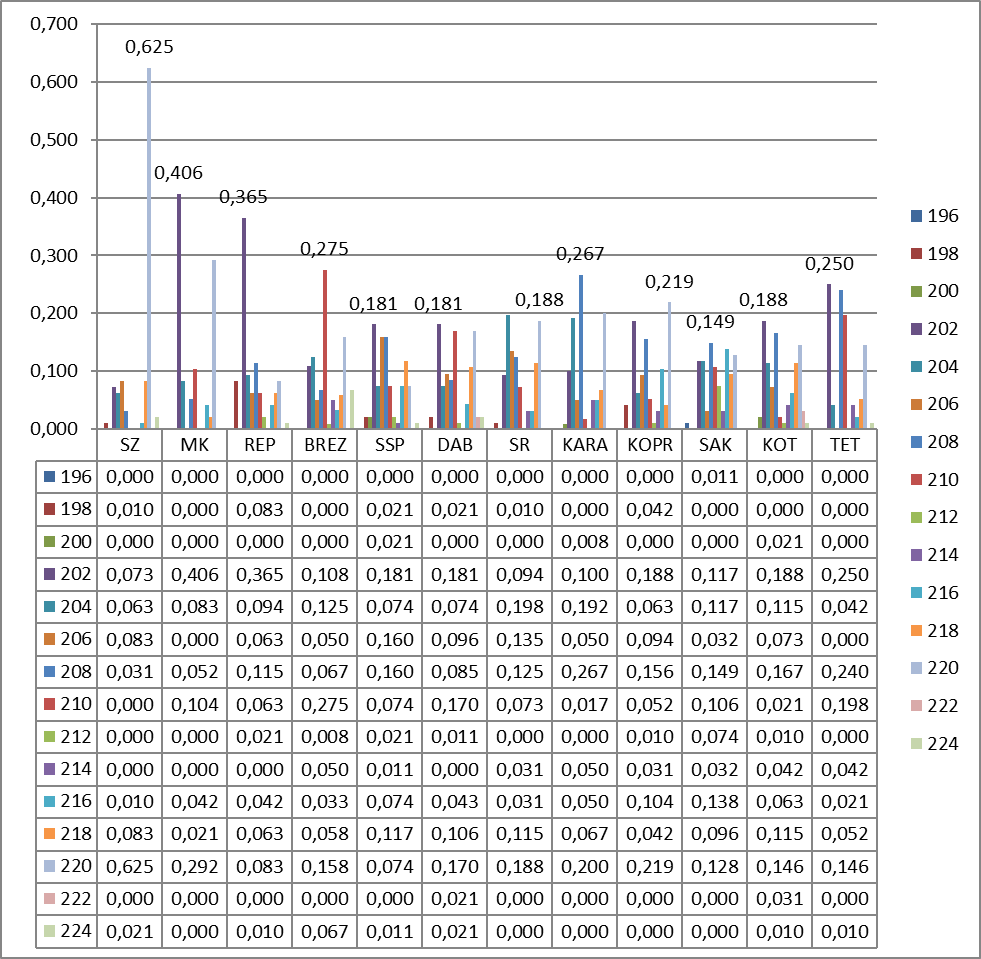


**Locus OarFCB20**


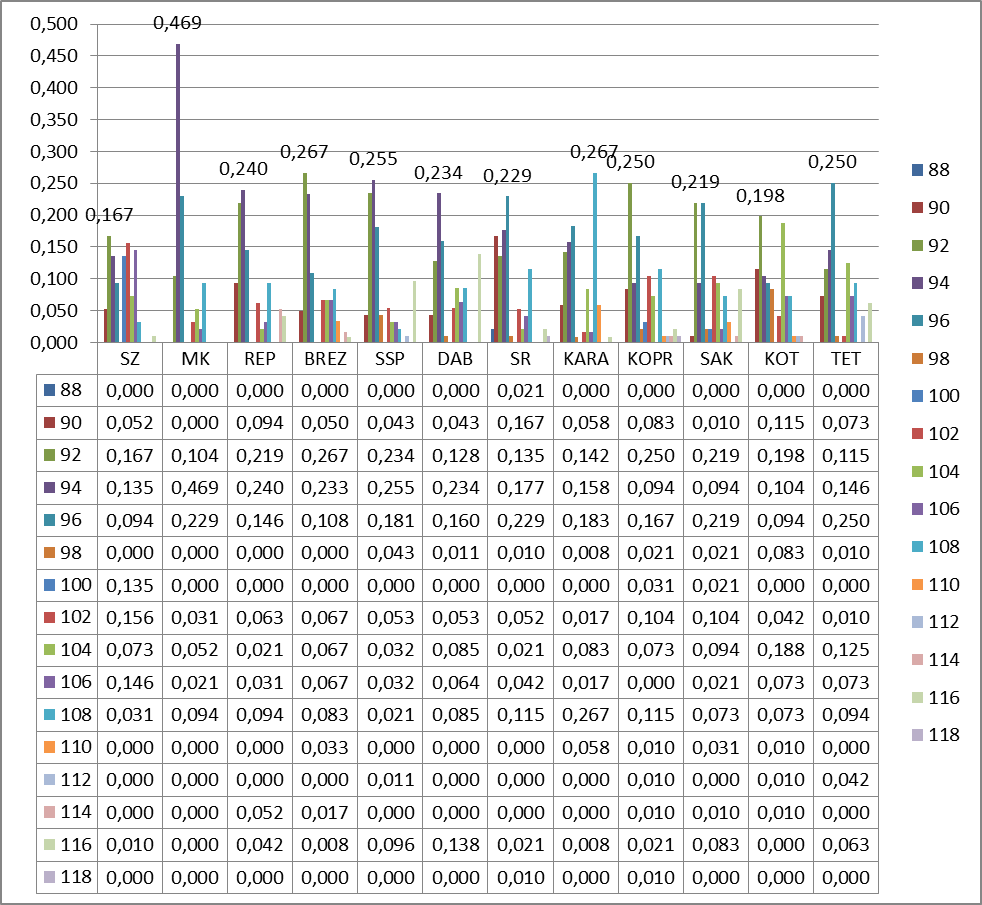


**Locus McM527**


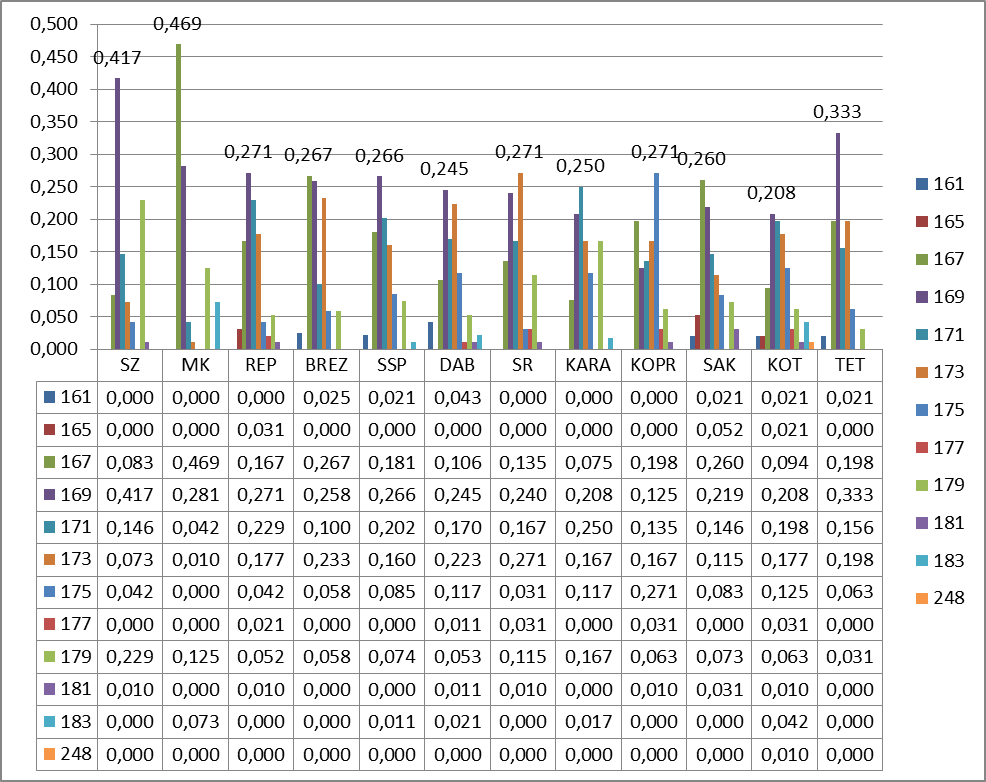


**Locus CSRD247**


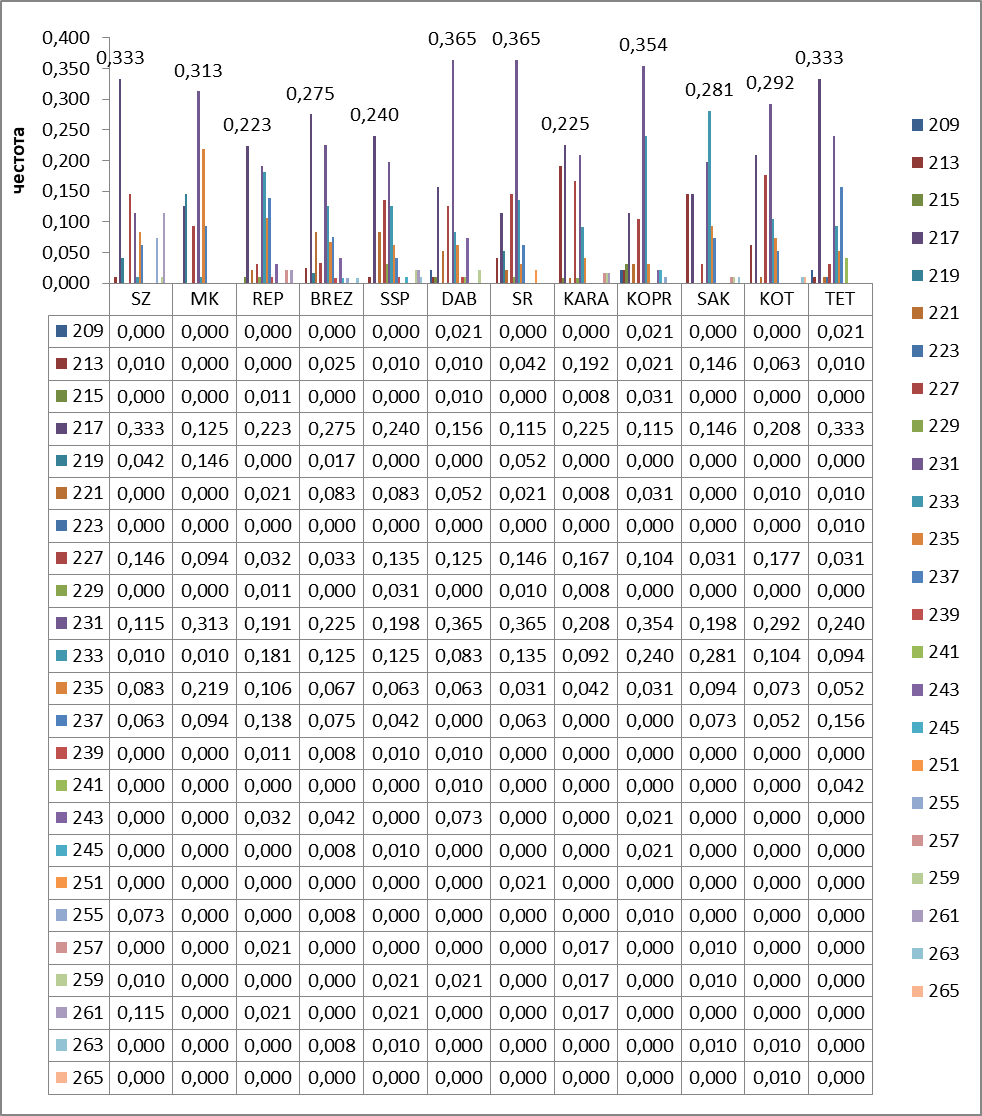


**Locus HSC**


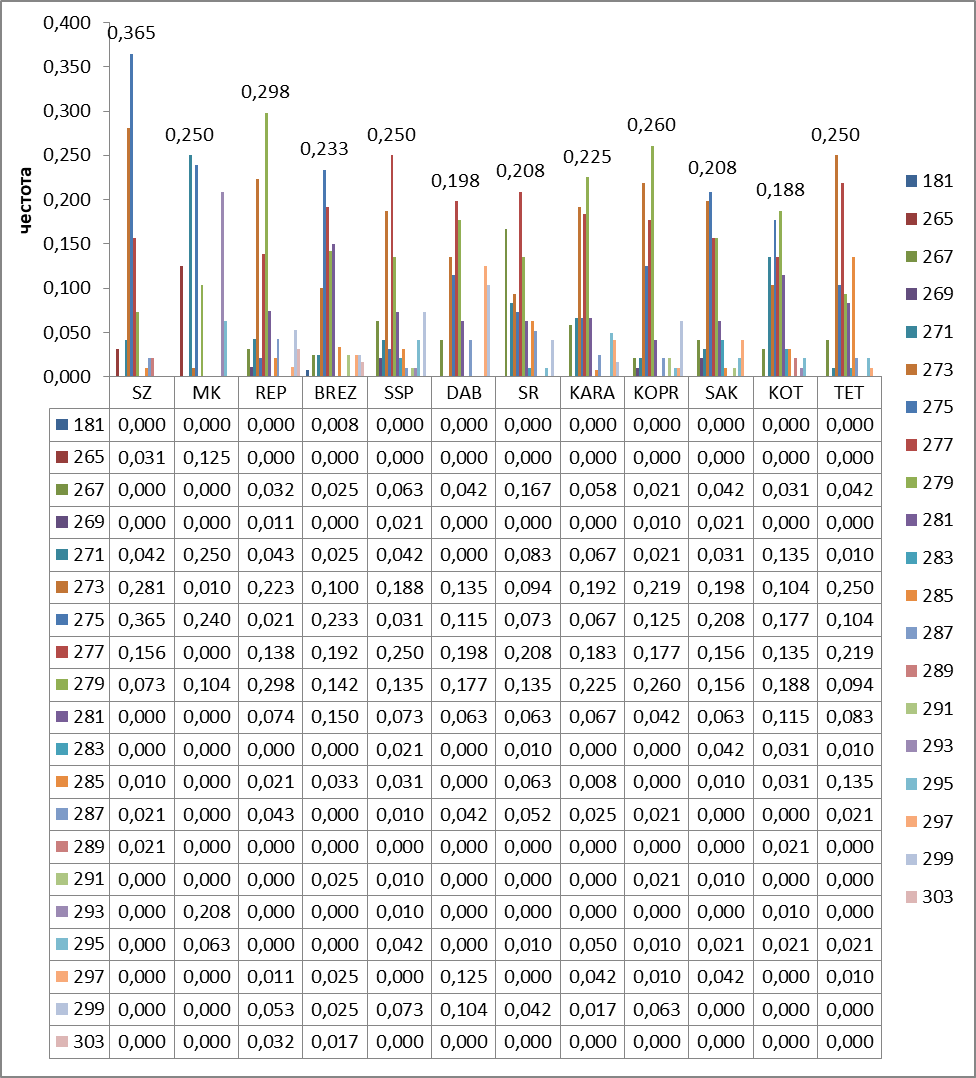


**Locus MAF214**


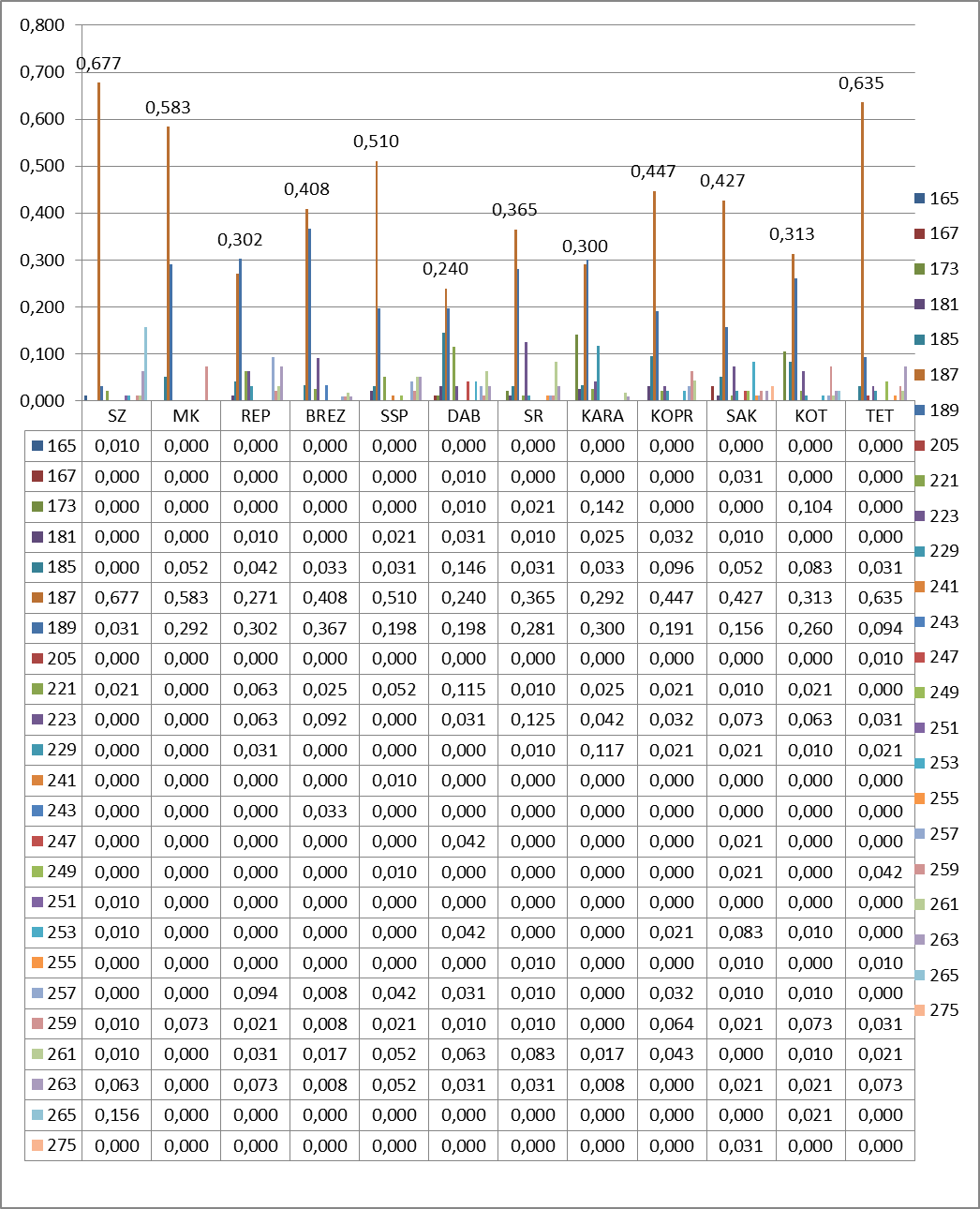


**Locus OarCP49**


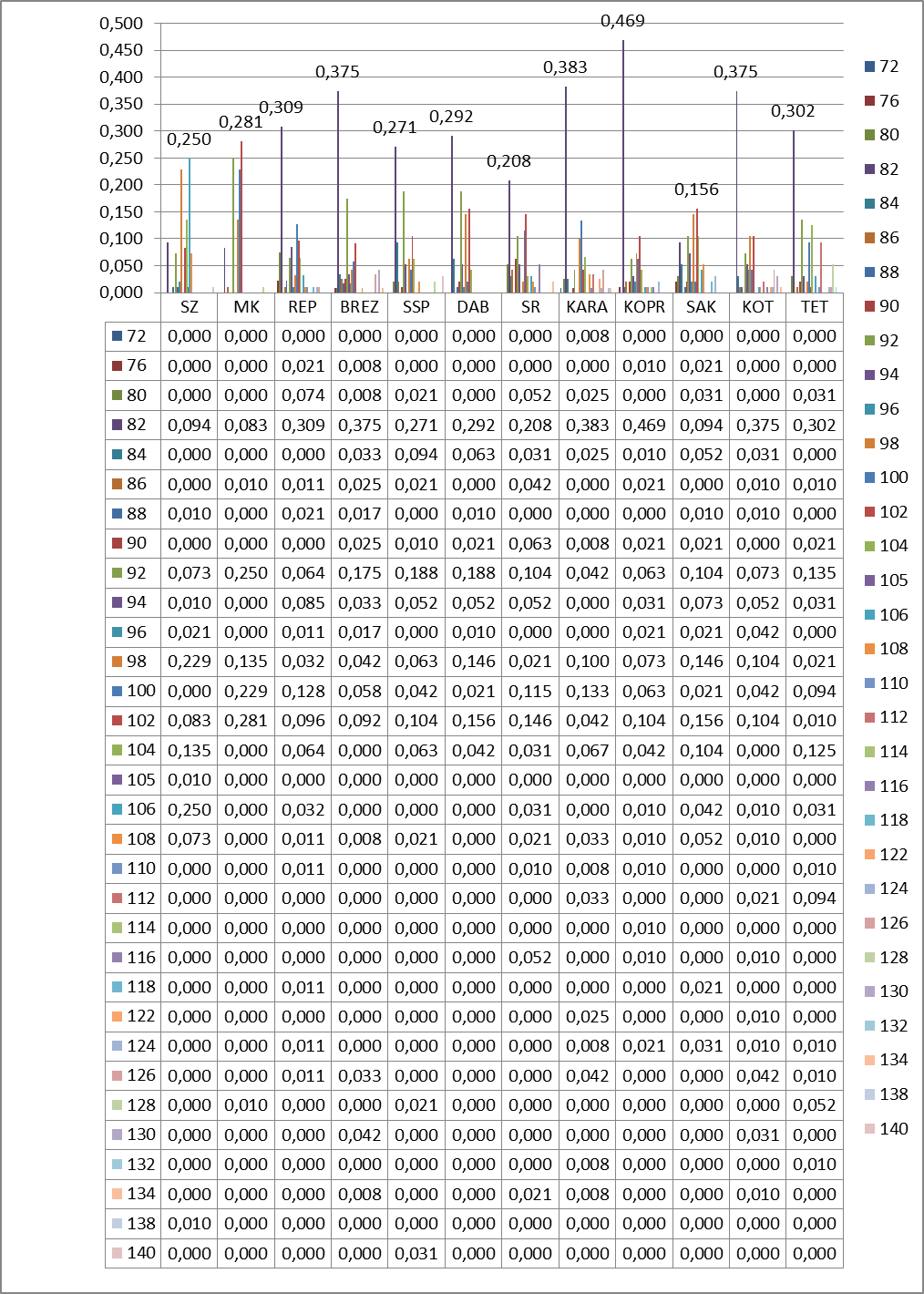


**Locus INRA63**


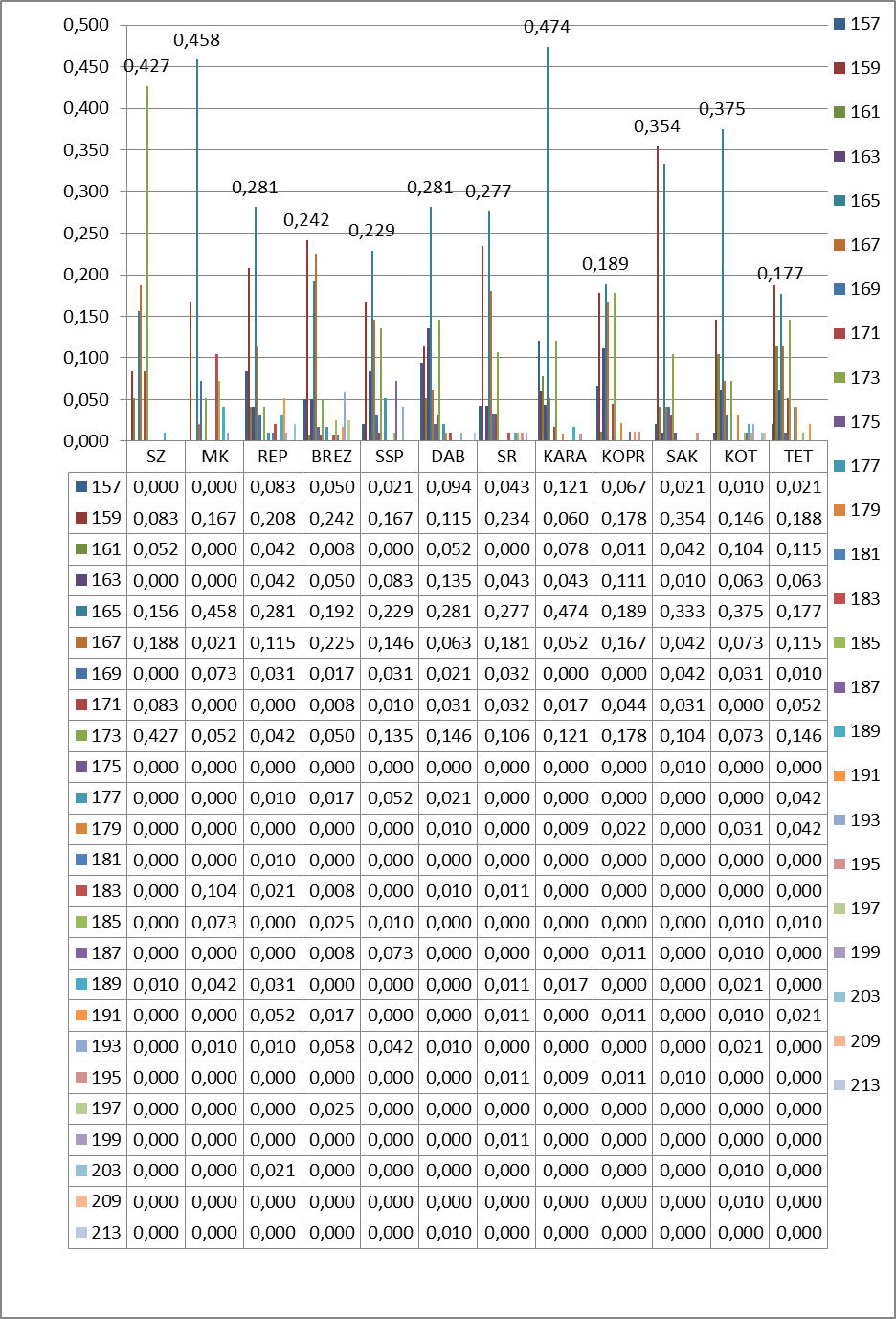

Supplement: Supplementary file 1 [file animals-13-01878-s001.zip › animals-2369886-supplementary/Supplementary Figure S1.docx]
