# Supplementary material for: Genetic Diversity and Population Structure of Bulgarian Autochthonous Sheep Breeds Revealed by Microsatellite Analysis
Source: Animals (Basel). 2023 Jun 5;13(11):1878. doi: 10.3390/ani13111878 (PMC10252131; doi:10.3390/ani13111878)
Supplement: Supplementary file 1 [file animals-13-01878-s001.zip › animals-2369886-supplementary/Supplementary Table S1.docx]

**Supplementary Table S1**. Distribution of flocks of breeds across the country. Some of the flocks are distributed outside of their natural habitats.

| **Flock** | **Breed** | **Town/Village** | **Municipality** | **Province** |
| --- | --- | --- | --- | --- |
| FL 1; FL 2 | SZ | vill. Gorno Botevo | Stara Zagora | Stara Zagora |
| FL 3 | SZ | twn Chirpan | Chirpan | Stara Zagora |
| FL 4 | SZ | twn Merichleri | Dimitrovgrad | Haskovo |
| FL 5 | MK | twn Karnobat | Karnobat | Burgas |
| FL 6 | MK | vill. Nevestino | Karnobat | Burgas |
| FL 7 | MK | twn Aytos | Aytos | Burgas |
| FL 8 | MK | twn Vetrino | Vetrino | Varna |
| FL 9; FL 10 | REP | twn Chiprovtsi | Chiprovtsi | Montana |
| FL 11 | REP | vill. Dabravka | Belogradchik | Vidin |
| FL 12 | REP | vill. Replyana | Chuprene | Vidin |
| FL 13 | BREZ | vill. Nepraznentsi | Breznik | Pernik |
| FL 14 | BREZ | vill. Slivnitsa | Kresna | Blagoevgrad |
| FL 15; FL 16 | BREZ | vill. Leskovets | Pernik | Pernik |
| FL 17 | BREZ | vill. Pozharevo | Bozhurishte | Sofia |
| FL 18 | SSP | vill. Gorni Domlyan | Karlovo | Plovdiv |
| FL 19 | SSP | twn Kalofer | Karlovo | Plovdiv |
| FL 20 | SSP | twn Apriltsi | Apriltsi | Lovech |
| FL 21 | SSP | vill. Mechkovitsa | Gabrovo | Gabrovo |
| FL 22 | DAB | vill. Chavdar | Chavdar | Sofia |
| FL 23; FL 24 | DAB | twn Karlovo | Karlovo | Plovdiv |
| FL 25 | DAB | vill. Dabene | Karlovo | Plovdiv |
| FL 26 | SR | vill. Borino | Borino | Smolyan |
| FL 27; FL 28 | SR | vill. Galabovo | Banite | Smolyan |
| FL 29 | SR | vill. Draganovo | Kirkovo | Kardzali |
| FL 30 | KARA | vill. Topolovo | Asenovgrad | Plovdiv |
| FL 31 | KARA | vill. Vlahi | Kresna | Blagoevgrad |
| FL 32 | KARA | vill. Postnik | Momchilgrad | Kardzali |
| FL 33 | KARA | vill. Vlahi | Kresna | Blagoevgrad |
| FL 34 | KARA | twn Smolyan | Smolyan | Smolyan |
| FL 35; FL 36; FL  37; FL 38 | KOPR | twn Koprivshtitsa | Koprivshtitsa | Sofia |
| FL 39 | SAK | vill. Kostandovo | Rakitovo | Pazardzhik |
| FL 40 | SAK | vill. Hlyabovo | Topolovgrad | Haskovo |
| FL 41 | SAK | vill. Oreshnik | Topolovgrad | Haskovo |
| FL 42 | SAK | twn Topolovgrad | Topolovgrad | Haskovo |
| FL 43 | KOT | vill. Topolchani | Sliven | Sliven |
| FL 44 | KOT | vill. Ichera | Sliven | Sliven |
| FL 45 | KOT | twn Kotel | Kotel | Sliven |
| FL 46 | KOT | vill. Mustrak | Svilengrad | Haskovo |
| FL 47; FL 48; FL  49; FL 50 | TET | twn Teteven | Teteven | Lovech |

Abbreviations: twn=town, vill.=village
