# Supplementary material for: Genetic Diversity and Population Structure of Bulgarian Autochthonous Sheep Breeds Revealed by Microsatellite Analysis
Source: Animals (Basel). 2023 Jun 5;13(11):1878. doi: 10.3390/ani13111878 (PMC10252131; doi:10.3390/ani13111878)
Supplement: Supplementary file 1 [file animals-13-01878-s001.zip › animals-2369886-supplementary/Supplementary Table S2.docx]

**Supplementary Table S2**. Information for the used microsatellite markers and their inclusion in multiplex PCR reactions. na= data not available.

| **STR marker** | **Chromosome location** | **Origin** | **Primer sequences F / R** | **Fw primer label** | **Multiplex PCR set** | **Allele range*** |
| --- | --- | --- | --- | --- | --- | --- |
| **CSRD247** | **14** | Ovine | GGACTTGCCAGAACTCTGCAAT | VIC | D | 209-265 |
|  |  |  | CACTGTGGTTTGTATTAGTCAGG |  |  |  |
| **HSC** | **20** | na | CTGCCAATGCAGAGACACAAGA | FAM | D | 267-297 |
|  |  |  | GTCTGTCTCCTGTCTTGTCATC |  |  |  |
| **INRA0063** | **14** | Bovine | ATTTGCACAAGCTAAATCTAACC | FAM | not included in a multiplex | 157-209 |
|  |  |  | AAACCACAGAAATGCTTGGAAG |  |  |  |
| **MAF0214** | **16** | Ovine | GGGTGATCTTAGGGAGGTTTTGGAGG | PET | D | 173-265 |
|  |  |  | AATGCAGGAGATCTGAGGCAGGGACG |  |  |  |
| **OarAE0129** | **5** | Ovine | AATCCAGTGTGTGAAAGACTAATCCAG | PET | B | 139-169 |
|  |  |  | GTAGATCAAGATATAGAATATTTTTCAACACC |  |  |  |
| **OarCP0049** | **17** | Ovine | CAGACACGGCTTAGCAACTAAACGC | VIC | D | 80-134 |
|  |  |  | GTGGGGATGAATATTCCTTCATAAGG |  |  |  |
| **OarFCB0011** | **2** | Ovine | GCAAGCAGGTTCTTTACCACTAGCACC | FAM | B | 122-144 |
|  |  |  | GGCCTGAACTCACAAGTTGATATATCTATCAC |  |  |  |
| **D5S2** | **5** | na | TACTCGTAGGGCAGGCTGCCTG | FAM | A | 188-202 |
|  |  |  | GAGACCTCAGGGTTGGTGATCAG |  |  |  |
| **INRA0005** | **12** | Bovine | TTCAGGCATACCCTACACCACATG | VIC | A | 116-152 |
|  |  |  | AAATATTAGCCAACTGAAAACTGGG |  |  |  |
| **INRA0023** | **3** | Bovine | GAGTAGAGCTACAAGATAAACTTC | PET | C | 202-224 |
|  |  |  | TAACTACAGGGTGTTAGATGAACTC |  |  |  |
| **MAF0065** | **15** | Ovine | AAAGGCCAGAGTATGCAATTAGGAG | PET | A | 125-143 |
|  |  |  | CCACTCCTCCTGAGAATATAACATG |  |  |  |
| **McM0527** | **5** | na | GTCCATTGCCTCAAATCAATT C | VIC | C | 161-248 |
|  |  |  | AAACCACTTGACTACTCCCCAA |  |  |  |
| **OarFCB0020** | **2** | Ovine | AAATGTGTTTAAGATTCCATACATGTG | FAM | C | 90-116 |
|  |  |  | GGAAAACCCCCATATATACCTATAC |  |  |  |

*Allele range according to literature data
