# Supplementary material for: Genetic Diversity and Population Structure of Bulgarian Autochthonous Sheep Breeds Revealed by Microsatellite Analysis
Source: Animals (Basel). 2023 Jun 5;13(11):1878. doi: 10.3390/ani13111878 (PMC10252131; doi:10.3390/ani13111878)
Supplement: Supplementary file 1 [file animals-13-01878-s001.zip › animals-2369886-supplementary/Supplementary Table S3.docx]

**Supplementary Table S3**. Hardy-Weinberg (HW) equilibrium test in the analyzed 13 microsatellite loci by breed.

| **Locus/ Breed** | **D5S2** | **INRA5** | **MAF65** | **OarAE129** | **OarFCB11** | **INRA23** | **OarFCB20** | **McM527** | **CSRD247** | **HSC** | **MAF214** | **OarCP49** | **INRA63** |
| --- | --- | --- | --- | --- | --- | --- | --- | --- | --- | --- | --- | --- | --- |
| **SZ** | 0.118 | 0.026* | 0.55 | 0.37 | 0.862 | 0.001*** | 0.017* | 0.604 | 0.355 | 0.003** | 1 | 0.759 | 0.522 |
| **MK** | 0.537 | 0.257 | 0.424 | 0.633 | 0.769 | 0.872 | 0.009** | 0.105 | 0.859 | 0.988 | 0.195 | 0.177 | 0.392 |
| **REP** | 0.001*** | 0.032* | 0.848 | 0*** | 0.302 | 0.739 | 0.564 | 0.657 | 0.789 | 0.009** | 0.65 | 0.956 | 0.314 |
| **BREZ** | 0.427 | 0.157 | 0.488 | 0.555 | 0.035* | 0.077 | 0.057 | 0.002** | 0.446 | 0.971 | 0.227 | 0*** | 0.95 |
| **SSP** | 0.4 | 0.684 | 0.008** | 0.999 | 0.756 | 0.015* | 0.474 | 0.145 | 0.187 | 0.203 | 0.264 | 0.128 | 0.216 |
| **DAB** | 0.001*** | 0.819 | 0.952 | 0.109 | 0.9 | 0.634 | 0.343 | 0.635 | 0.985 | 0.011* | 0.2 | 0.98 | 0.992 |
| **SR** | 0.598 | 0.83 | 0.215 | 0.002** | 0.911 | 0.032* | 0.086 | 0.393 | 0.898 | 0.433 | 0.966 | 0.521 | 0.341 |
| **KARA** | 0.052 | 0.004** | 0.015* | 0.005** | 0.108 | 0.015* | 0.346 | 0.071 | 0.979 | 0.855 | 0.248 | 0.723 | 0.267 |
| **KOPR** | 0.711 | 0.001*** | 0.002** | 0.256 | 0.91 | 0.255 | 0.01** | 0.741 | 0.703 | 0.078 | 0.177 | 0.013* | 0.947 |
| **SAK** | 0.755 | 0.441 | 0.012* | 0.615 | 0.522 | 0.397 | 0.871 | 0.947 | 0.825 | 0.946 | 0.017* | 0.598 | 0.993 |
| **KOT** | 0.432 | 0.456 | 0.998 | 0.847 | 0.876 | 0.865 | 0.437 | 1 | 0*** | 0.427 | 0.834 | 0.999 | 0,393 |
| **ТЕТ** | 0.025* | 0.618 | 0.982 | 0.424 | 0.625 | 0.667 | 0.731 | 0*** | 0.001*** | 0*** | 1 | 1 | 0.037 |

Significant *p* values: ****p*<0.05, ***p*<0.01, ****p*<0.001**
