# Supplementary material for: Genetic Diversity and Population Structure of Bulgarian Autochthonous Sheep Breeds Revealed by Microsatellite Analysis
Source: Animals (Basel). 2023 Jun 5;13(11):1878. doi: 10.3390/ani13111878 (PMC10252131; doi:10.3390/ani13111878)
Supplement: Supplementary file 1 [file animals-13-01878-s001.zip › animals-2369886-supplementary/Supplementary Table S4.docx]

**Supplemental Table S4.** Genetic distances between breeds according to Nei.

| **Breed** | **SZ** | **MK** | **REP** | **BREZ** | **SSP** | **DAB** | **SR** | **KARA** | **KOPR** | **SAK** | **KOT** |
| --- | --- | --- | --- | --- | --- | --- | --- | --- | --- | --- | --- |
| **MK** | 0.362 |  |  |  |  |  |  |  |  |  |  |
| **REP** | 0.412 | 0.233 |  |  |  |  |  |  |  |  |  |
| **BREZ** | 0.294 | 0.257 | 0.108 |  |  |  |  |  |  |  |  |
| **SSP** | 0.325 | 0.243 | 0.067 | 0.063 |  |  |  |  |  |  |  |
| **DAB** | 0.316 | 0.229 | 0.068 | 0.078 | 0.071 |  |  |  |  |  |  |
| **SR** | 0.308 | 0.209 | 0.066 | 0.090 | 0.062 | 0.065 |  |  |  |  |  |
| **KARA** | 0.437 | 0.312 | 0.109 | 0.167 | 0.108 | 0.133 | 0.090 |  |  |  |  |
| **KOPR** | 0.316 | 0.278 | 0.095 | 0.106 | 0.086 | 0.079 | 0.083 | 0.139 |  |  |  |
| **SAK** | 0.300 | 0.202 | 0.102 | 0.130 | 0.094 | 0.098 | 0.075 | 0.162 | 0.114 |  |  |
| **KOT** | 0.349 | 0.248 | 0.061 | 0.088 | 0.057 | 0.063 | 0.052 | 0.044 | 0.083 | 0.099 |  |
| **TET** | 0.362 | 0.282 | 0.107 | 0.131 | 0.079 | 0.130 | 0.107 | 0.181 | 0.106 | 0.130 | 0.113 |
